# Supplementary material for: Role of individual and population heterogeneity in shaping dynamics of multi-pathogen shedding in an island endemic bat
Source: PLoS Pathog. 2025 Jul 11;21(7):e1013334. doi: 10.1371/journal.ppat.1013334 (PMC12273948; doi:10.1371/journal.ppat.1013334)
Supplement: S1 Table — Significant variables are in bold and the asterisk represents the interaction between two variables. All GAMs were fitted with a binomial distribution. Model M4 (GLM) was fitted with a Gaussian distribution, and its performance was compared to a null model using AIC criterion. The percentage of deviance explained was calculated by comparing full model with null model. PMV: Paramyxovirus, LEPTO: Leptospira bacteria, HSV: Herpesvirus. (DOCX) [file ppat.1013334.s001.docx]

**S1 Table. Summary of the statistical models (models M1 to M4) used to analyse single shedding dynamics in *M. francoismoutoui.*** Significant variables are in bold and the asterisk represents the interaction between two variables. All GAMs were fitted with a binomial distribution. Model M4 (GLM) was fitted with a Gaussian distribution, and its performance was compared to a null model using AIC criterion. The percentage of deviance explained was calculated by comparing full model with null model. PMV: Paramyxovirus, LEPTO: *Leptospira* bacteria, HSV: Herpesvirus.

| *Type and model number* | *Levels and number of individuals* | *Response variable* | *Deviance explained*  *(%)* | *Explanatory variables* | *EDF* | *Chi²* | *Estimate (± SE)* | *Odds ratios* | *Z value* | *P* |
| --- | --- | --- | --- | --- | --- | --- | --- | --- | --- | --- |
| GAM  M1 | All individuals  N = 5518 | PMV | 14.1 | **s(Sampling period)**  s(SSAS)  logSize  **Age**  **Sex**  Age*Sex  **LEPTO**  **HSV**  **LEPTO*HSV** | 5.70  4.32 | 15.05  1.55 | 0.01 (±0.03)  -3.46 (±0.59)  -0.45 (±0.09)  0.99 (±0.75)  2.54 (±0.64)  2.65 (±0.52)  -2.00 (±0.65) | 1.01  0.03  0.64  2.70  12.62  14.13  0.14 | 0.37  -5.83  -5.03  1.33  3.96  5.12  -3.10 | 0.032  0.90  0.71  5.62^-09^  4.81^-07^  0.18  7.47^-05^  2.99^-07^  0.002 |
| GAM  M2 | All individuals  N = 5518 | LEPTO | 10.3 | s(Sampling period)  s(SSAS)  logSize  **Age**  Sex  Age*Sex  **PMV**  **HSV**  **PMV*HSV** | 6.17  2.89 | 12.66  1.76 | 0.02 (±0.03)  -1.41 (±0.23)  -0.07 (±0.09)  0.04 (±0.29)  2.09 (±0.62)  1.12 (±0.19)  -1.54 (±0.62) | 1.02  0.24  0.94  1.04  8.10  3.05  0.21 | 0.80  -6.17  -0.77  0.14  3.35  5.89  -2.46 | 0.09  0.60  0.43  6.73^-10^  0.44  0.89  7.99^-04^  3.95^-09^  0.01 |
| GAM  M3 | All individuals  N = 3981 | HSV | 44.9 | **s(Sampling period)**  s(SSAS)  **logSize**  **Age**  **Sex**  **Age*Sex**  **PMV**  **LEPTO**  **PMV*LEPTO** | 6.67  4.93 | 19.86  7.38 | -0.14 (±0.05)  -2.80 (±0.23)  0.81 (±0.21)  -0.88 (±0.29)  2.52 (±0.52)  1.09 (±0.19)  1.59 (±0.63) | 0.87  0.06  2.25  0.41  12.47  2.98  0.20 | -2.83  11.88  3.84  -3.03  4.84  5.63  -2.51 | 0.01  0.40  0.005  2^-16^  1.25^-04^  0.002  1.30^-06^  1.81^-08^  0.01 |
| GAM  M3bis | Adults only  N = 3344 | HSV | 14.8 | s(Sampling period)  s(SSAS)  logSize  **Sex**  **PMV**  **LEPTO**  **PMV*LEPTO** | 5.90  4.59 | 11.37  1.57 | -0.08 (±0.09)  0.96 (±0.22)  2.30 (±0.52)  1.38 (±0.28)  -1.74 (±0.66) | 0.93  2.62  9.93  3.98  0.18 | -0.83  4.44  4.42  4.87  -2.63 | 0.14  0.93  0.41  9.06^-06^  9.75^-06^  1.12^-06^  0.009 |
| GLM  M4 | *Leptospira* PCR-positive individuals  N = 2529 | Ct LEPTO | 5.2 | **Age (AIC = 14036)**  Null (AIC = 14169) |  |  | 4.53 (±0.38) |  | 11.78 | 2^-16^ |
