# Supplementary material for: Role of individual and population heterogeneity in shaping dynamics of multi-pathogen shedding in an island endemic bat
Source: PLoS Pathog. 2025 Jul 11;21(7):e1013334. doi: 10.1371/journal.ppat.1013334 (PMC12273948; doi:10.1371/journal.ppat.1013334)
Supplement: S2 Table — GLMs were fitted with a binomial distribution, except for Ct LEPTO variable fitted with gaussian distribution. Final models (in bold) were selected by comparing full model with null model and using best AIC criterion (when ΔAIC > 2). The percentage of deviance explained was calculated by comparing full model with null model. PMV: Paramyxovirus, LEPTO: Leptospira bacteria, HSV: Herpesvirus. M0: female with no visible nipples. (DOCX) [file ppat.1013334.s002.docx]

**S2 Table. Summary of the statistical models (models M5 to M12) used to analyse the mono-excretion dynamics in *M. francoismoutoui* during pregnancy and mating periods, specifically.** GLMs were fitted with a binomial distribution, except for Ct LEPTO variable fitted with gaussian distribution. Final models (in bold) were selected by comparing full model with null model and using best AIC criterion (when ΔAIC>2). The percentage of deviance explained was calculated by comparing full model with null model. PMV: Paramyxovirus, LEPTO: *Leptospira* bacteria, HSV: Herpesvirus. M0: female with no visible nipples.

| *Type and model number* | *Levels and number of individuals* | *Deviance explained*  *(%)* | *Response variable* | *Explanatory variables (AIC)* | *Estimate (± SE)* | *Z value* | *P* |
| --- | --- | --- | --- | --- | --- | --- | --- |
| GLM  M5 | Adult females  N = 523 | 5.2 | PMV | **Pregnancy (684)**  Null (720) | 1.49 (±0.26) | 5.76 | 8.31^-09^ |
| GLM  M5bis | Adult females, without non-pregnant M0  N = 470 | 0.1 | PMV | Pregnancy (630)  **Null (628)** | 0.25 (±0.35) | 0.72 | 0.47 |
| GLM  M6 | Adult female  N = 522 | 4.1 | LEPTO | **Pregnancy (682)**  Null (709) | 1.27 (±0.25) | 5.20 | 2.02^-07^ |
| GLM  M6bis | Adult females, without non-pregnant M0  N = 469 | 0.3 | LEPTO | Pregnancy (618)  **Null (618)** | 0.47 (±0.35) | 1.35 | 0.18 |
| GLM  M7 | Adult females  N = 307 | 0.9 | Ct LEPTO | Pregnancy (1713)  **Null (1713)** | -1.27 (±0.75) | -1.69 | 0.09 |
| GLM  M7bis | Adult females, without non-pregnant M0  N = 296 | 0.01 | Ct LEPTO | Pregnancy (1637)  **Null (1635)** | 0.16 (±0.91) | 0.18 | 0.86 |
| GLM  M8 | Adult females  N = 377 | 1.4 | HSV | Pregnancy (102)  **Null (101)** | 0.78 (±0.64) | 1.22 | 0.22 |
| GLM  M8bis | Adult females, without non-pregnant M0  N = 331 | 2.2 | HSV | Pregnancy (70)  **Null (70)** | -15.84 (±1872) | -0.008 | 0.99 |
| GLM  M9 | Adult males  N = 353 | 0.001 | PMV | Reproduction (485)  **Null (483)** | 0.02 (±0.22) | 0.09 | 0.93 |
| GLM  M10 | Adult males  N = 353 | 1.5 | LEPTO | **Reproduction (483)**  Null (489) | 0.59 (±0.22) | 2.70 | 0.007 |
| GLM  M11 | Adult males  N = 163 | 1.6 | Ct LEPTO | Reproduction (898)  **Null (899)** | 0.96 (±0.59) | 1.62 | 0.11 |
| GLM  M12 | Adult males  N = 228 | 4.4 | HSV | Reproduction (63)  **Null (64)** | 1.55 (±1.09) | 1.42 | 0.16 |
