# Supplementary material for: Role of individual and population heterogeneity in shaping dynamics of multi-pathogen shedding in an island endemic bat
Source: PLoS Pathog. 2025 Jul 11;21(7):e1013334. doi: 10.1371/journal.ppat.1013334 (PMC12273948; doi:10.1371/journal.ppat.1013334)
Supplement: S3 Table — Significant variables are in bold and the asterisk represents the interaction between two variables. The percentage of deviance explained was calculated by comparing full model with null model. All GAMs were fitted with a binomial distribution. PMV: Paramyxovirus, LEPTO: Leptospira bacteria, HSV: Herpesvirus. (DOCX) [file ppat.1013334.s003.docx]

**S3 Table. Summary of the statistical models (models M13 to M16) used to analyse dual and triple shedding dynamics in *M. francoismoutoui*.** Significant variables are in bold and the asterisk represents the interaction between two variables. The percentage of deviance explained was calculated by comparing full model with null model. All GAMs were fitted with a binomial distribution. PMV: Paramyxovirus, LEPTO: *Leptospira* bacteria, HSV: Herpesvirus.

| *Type and model number* | *Levels and number of individuals* | *Response variable* | *Deviance explained*  *(%)* | *Explanatory variables* | *EDF* | *Chi²* | *Estimate (± SE)* | *Z value* | *P* |
| --- | --- | --- | --- | --- | --- | --- | --- | --- | --- |
| GAM  M13 | Individuals tested for the three infectious agents  N = 3784 | PMV - LEPTO | 9.1 | s(Sampling period)  s(SSAS)  logSize  **Age**  **Sex**  Age*Sex | 5.45  4.17 | 5.68  3.71 | 0.04 (±0.04)  -4.75 (±1.01)  -0.49 (±0.10)  0.46 (±1.42) | 1.02  -4.73  -5.04  0.33 | 0.52  0.67  0.31  2.30^-06^  4.70^-07^  0.75 |
| GAM  M14 | Individuals tested for the three infectious agents  N = 3784 | LEPTO - HSV | 10.8 | s(Sampling period)  s(SSAS)  logSize  **Age**  Sex  Age*Sex | 5.81  4.39 | 11.67  2.91 | 0.02 (±0.03)  -2.81 (±0.26)  -0.06 (±0.08)  -0.26 (±0.39) | 0.52  -10.67  -0.67  -0.67 | 0.14  0.71  0.61  2^-16^  0.51  0.50 |
| GAM  M15 | Individuals tested for the three infectious agents  N = 3784 | PMV -HSV | 11.0 | **s(Sampling period)**  s(SSAS)  logSize  **Age**  **Sex**  Age*Sex | 5.74  4.34 | 17.46  1.94 | 0.007 (±0.03)  -4.33 (±0.59)  -0.37 (±0.09)  0.88 (±0.74) | 0.22  -7.39  -4.26  1.19 | 0.01  0.86  0.83  1.43^-13^  2.01^-05^  0.24 |
| GAM  M16 | Individuals tested for the three infectious agents  N = 3784 | PMV – LEPTO - HSV | 8.9 | s(Sampling period)  s(SSAS)  logSize  **Age**  **Sex**  Age*Sex | 5.44  4.17 | 5.33  4.21 | 0.04 (±0.04)  -4.72 (±1.01)  -0.47 (±0.10)  0.44 (±1.42) | 1.00  -4.69  -4.76  0.31 | 0.56  0.62  0.32  2.68^-06^  1.98^-06^  0.76 |
