# Supplementary material for: Role of individual and population heterogeneity in shaping dynamics of multi-pathogen shedding in an island endemic bat
Source: PLoS Pathog. 2025 Jul 11;21(7):e1013334. doi: 10.1371/journal.ppat.1013334 (PMC12273948; doi:10.1371/journal.ppat.1013334)
Supplement: S4 Table — GLMs were fitted with a binomial distribution. Final models (in bold) were selected by comparing full model with null model and using best AIC criterion (when ΔAIC > 2). The percentage of deviance explained was calculated by comparing full model with null model. PMV: Paramyxovirus, LEPTO: Leptospira bacteria, HSV: Herpesvirus. M0: female with no visible nipples. (DOCX) [file ppat.1013334.s004.docx]

**S4 Table.** **Summary of the statistical models (models M17 to M24) used to analyse dual and triple shedding dynamics in *M. francoismoutoui* during pregnancy and mating periods specifically.** GLMs were fitted with a binomial distribution. Final models (in bold) were selected by comparing full model with null model and using best AIC criterion (when ΔAIC>2). The percentage of deviance explained was calculated by comparing full model with null model. PMV: Paramyxovirus, LEPTO: *Leptospira* bacteria, HSV: Herpesvirus. M0: female with no visible nipples.

| *Type and model number* | *Levels and number of individuals* | *Response variable* | *Deviance explained (%)* | *Explanatory variables (AIC)* | *Estimate (± SE)* | *Z value* | *P* |
| --- | --- | --- | --- | --- | --- | --- | --- |
| GLM  M17 | Adult females  N = 370 | PMV - LEPTO | 4.3 | **Pregnancy (456)**  Null (474) | 1.39 (±0.35) | 4.01 | 6.15^-05^ |
| GLM  M17bis | Adult females, without non-pregnant M0  N = 323 | PMV - LEPTO | 0.2 | Pregnancy (432)  **Null (431)** | 0.38 (±0.40) | 0.97 | 0.33 |
| GLM  M18 | Adult females  N = 370 | LEPTO - HSV | 5.2 | **Pregnancy (480)**  Null (504) | 1.32 (±0.27) | 4.93 | 8.05^-07^ |
| GLM  M18bis | Adult females, without non-pregnant M0  N = 323 | LEPTO - HSV | 0.4 | Pregnancy (423)  **Null (423)** | 0.46 (±0.37) | 1.24 | 0.22 |
| GLM  M19 | Adult females  N = 370 | PMV - HSV | 5.6 | **Pregnancy (487)**  Null (514) | 1.42 (±0.28) | 5.07 | 3.97^-07^ |
| GLM  M19bis | Adult females without non-pregnant M0  N = 323 | PMV - HSV | 0.1 | Pregnancy (441)  **Null (439)** | 0.21 (±0.37) | 0.57 | 0.57 |
| GLM  M20 | Adult females  N = 370 | PMV - LEPTO - HSV | 3.8 | **Pregnancy (451)**  Null (467) | 1.31 (±0.35) | 3.79 | 1.5^-04^ |
| GLM  M20bis | Adult females, without non-pregnant M0  N = 323 | PMV - LEPTO -HSV | 0.1 | Pregnancy (427)  **Null (426)** | 0.31 (±0.40) | 0.78 | 0.43 |
| GLM  M21 | Adult males  N = 214 | PMV - LEPTO | 0.3 | Reproduction (231)  **Null (230)** | 0.29 (±0.33) | 0.89 | 0.38 |
| GLM  M22 | Adult males  N = 214 | LEPTO - HSV | 2.4 | **Reproduction (293)**  Null (298) | 0.75 (±0.28) | 2.66 | 0.008 |
| GLM  M23 | Adult males  N = 214 | PMV -HSV | 0.1 | Reproduction (297)  **Null (295)** | -0.18 (±0.28) | -0.63 | 0.53 |
| GLM  M24 | Adult males  N = 214 | PMV – LEPTO - HSV | 0.3 | Reproduction (231)  **Null (230)** | 0.29 (±0.33) | 0.89 | 0.38 |
