# Supplementary material for: Role of individual and population heterogeneity in shaping dynamics of multi-pathogen shedding in an island endemic bat
Source: PLoS Pathog. 2025 Jul 11;21(7):e1013334. doi: 10.1371/journal.ppat.1013334 (PMC12273948; doi:10.1371/journal.ppat.1013334)
Supplement: S5 Table — Transitions in Herpesvirus shedding status was not analysed because not enough negative saliva samples were available. Interval: time interval (days) between recaptures. Repro: reproductive status transition (active to active, non-active to active, active to non-active and non-active to non-active). PMV: Paramyxovirus, LEPTO: Leptospira bacteria. (DOCX) [file ppat.1013334.s005.docx]

**S5 Table. Summary of the statistical models (models M25 and M26) used to analyze recapture data in *M. francoismoutoui*.** Transitions in Herpesvirus shedding status was not analysed because not enough negative saliva samples were available. Interval: time interval (days) between recaptures. Repro: reproductive status transition (active to active, non-active to active, active to non-active and non-active to non-active). PMV: Paramyxovirus, LEPTO: *Leptospira* bacteria.

| *Type and model number* | *Levels and number of individuals* | *Response variable* | *Explanatory variables* | *AIC* |
| --- | --- | --- | --- | --- |
| Multinomial  M25 (a-h) | All individuals recaptured at least one time  N = 457 | LEPTO | a: Interval + Sex + Repro  b: Sex + Repro  c: Interval + Sex  d: **Interval + Repro**  e: Sex  f: Repro  g: Interval  h: Null | 1145  1157  1144  1140  1155  1152  1142  1153 |
| Multinomial  M26 (a-h) | All individuals recaptured at least one time  N = 471 | PMV | a: Interval + Sex + Repro  b: Sex + Repro  c: Interval + Sex  d: Interval + Repro  e: Sex  f: **Repro**  g: Interval  h: Null | 1123  1122  1130  1119  1128  1118  1134  1132 |
