# Supplementary figures and images for: Role of individual and population heterogeneity in shaping dynamics of multi-pathogen shedding in an island endemic bat
Source: PLoS Pathog. 2025 Jul 11;21(7):e1013334. doi: 10.1371/journal.ppat.1013334 (PMC12273948; doi:10.1371/journal.ppat.1013334)

**S6 Table. Characteristics and coding of sampling periods used in GAMs.**

**
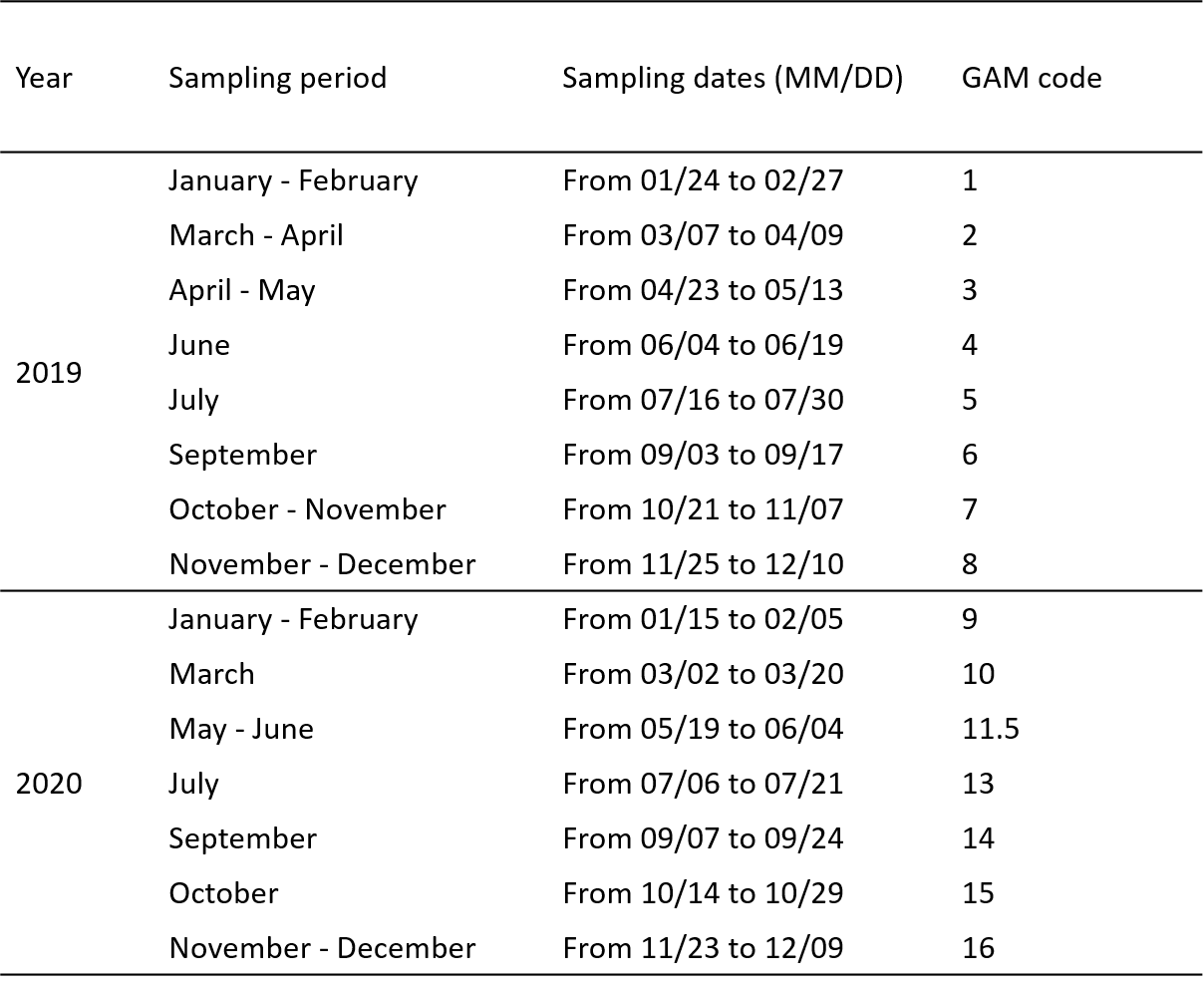
**

Supplement: S6 Table — (DOCX) [file ppat.1013334.s006.docx]
