# Supplementary material for: Role of individual and population heterogeneity in shaping dynamics of multi-pathogen shedding in an island endemic bat
Source: PLoS Pathog. 2025 Jul 11;21(7):e1013334. doi: 10.1371/journal.ppat.1013334 (PMC12273948; doi:10.1371/journal.ppat.1013334)
Supplement: S1 Fig — Note that in the green group, bats can include both adult bats that were not sexually mature yet, as well as misclassified juveniles. (DOCX) [file ppat.1013334.s007.docx]

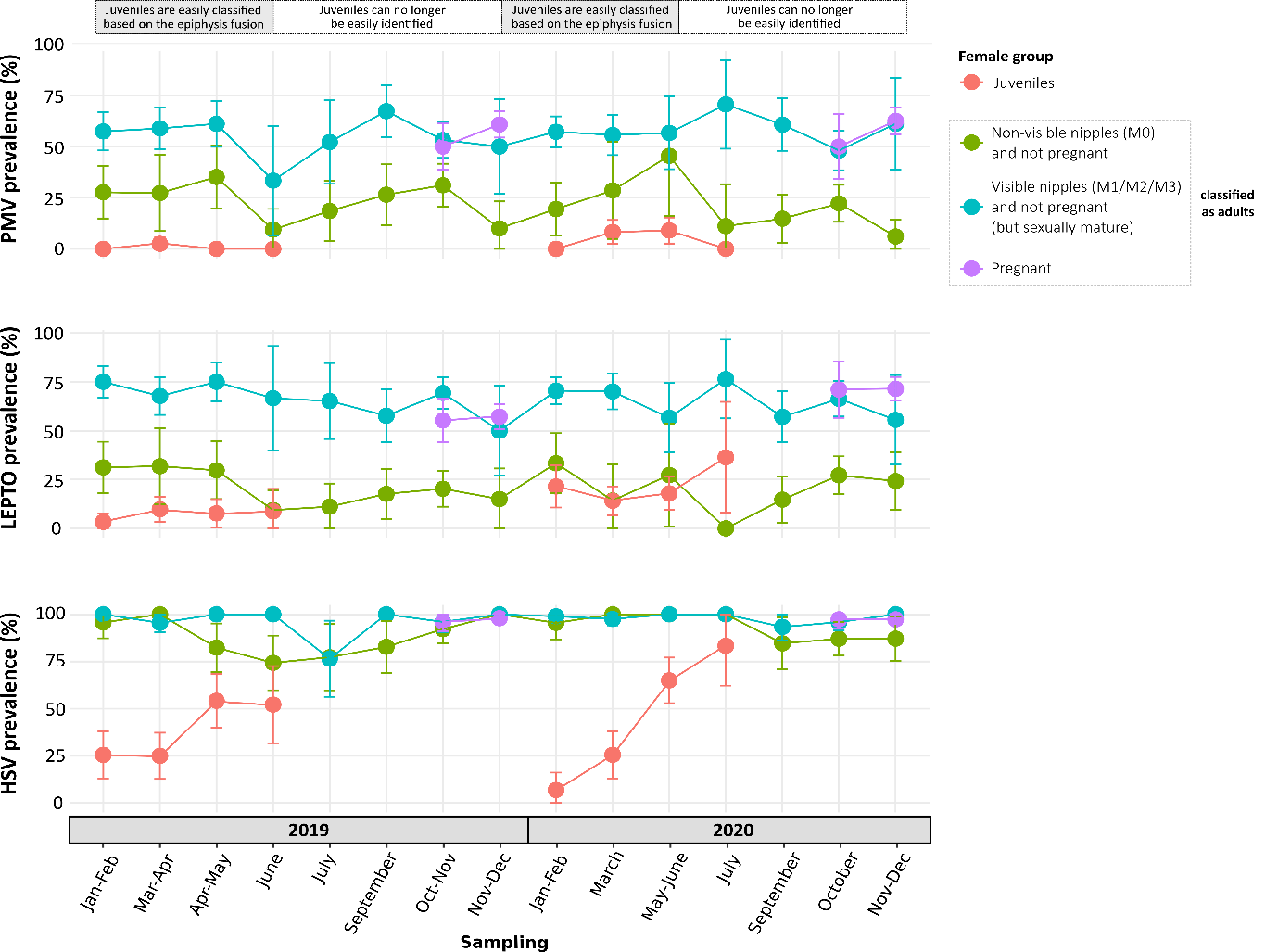


**S1 Fig.** **Temporal variation of paramyxovirus (PMV), *Leptospira* (LEPTO) and Herpesvirus (HSV) prevalence in *M. francoismoutoui* females, according to age and reproductive characteristics (pregnancy and visibility of nipples).** Note that in the green group, bats can include both adult bats that were not sexually mature yet, as well as misclassified juveniles.
