# Supplementary material for: Role of individual and population heterogeneity in shaping dynamics of multi-pathogen shedding in an island endemic bat
Source: PLoS Pathog. 2025 Jul 11;21(7):e1013334. doi: 10.1371/journal.ppat.1013334 (PMC12273948; doi:10.1371/journal.ppat.1013334)
Supplement: S2 Fig — Observed proportions are shown with 95% confidence intervals (CI), and expected prevalence and 95% CI are indicated below in square brackets. (DOCX) [file ppat.1013334.s008.docx]

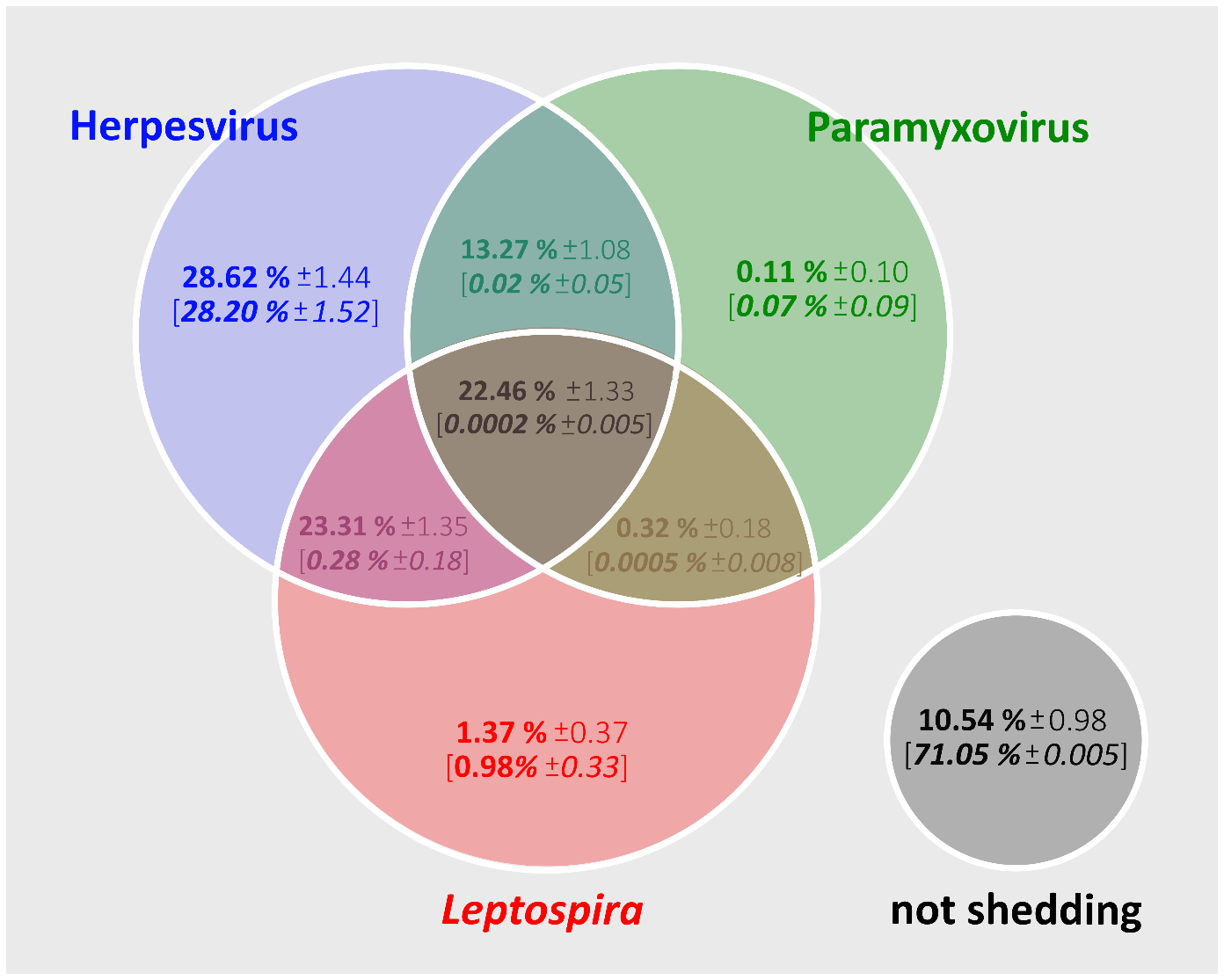


**S2 Fig. Venn diagram of *M. francoismoutoui* bats tested for the three infectious agents (n = 3784).** Observed proportions are shown with 95% confidence intervals (CI), and expected prevalence and 95% CI are indicated below in square brackets.
