# Supplementary material for: Role of individual and population heterogeneity in shaping dynamics of multi-pathogen shedding in an island endemic bat
Source: PLoS Pathog. 2025 Jul 11;21(7):e1013334. doi: 10.1371/journal.ppat.1013334 (PMC12273948; doi:10.1371/journal.ppat.1013334)
Supplement: S3 Fig — (A) Distribution of time interval between recaptures. (B) Variation of time intervals across the four categories of shedding status transitions, for Leptospira and paramyxovirus. Shedding status is coded with 0 for non-shedding and 1 for shedding bats. (DOCX) [file ppat.1013334.s009.docx]

**
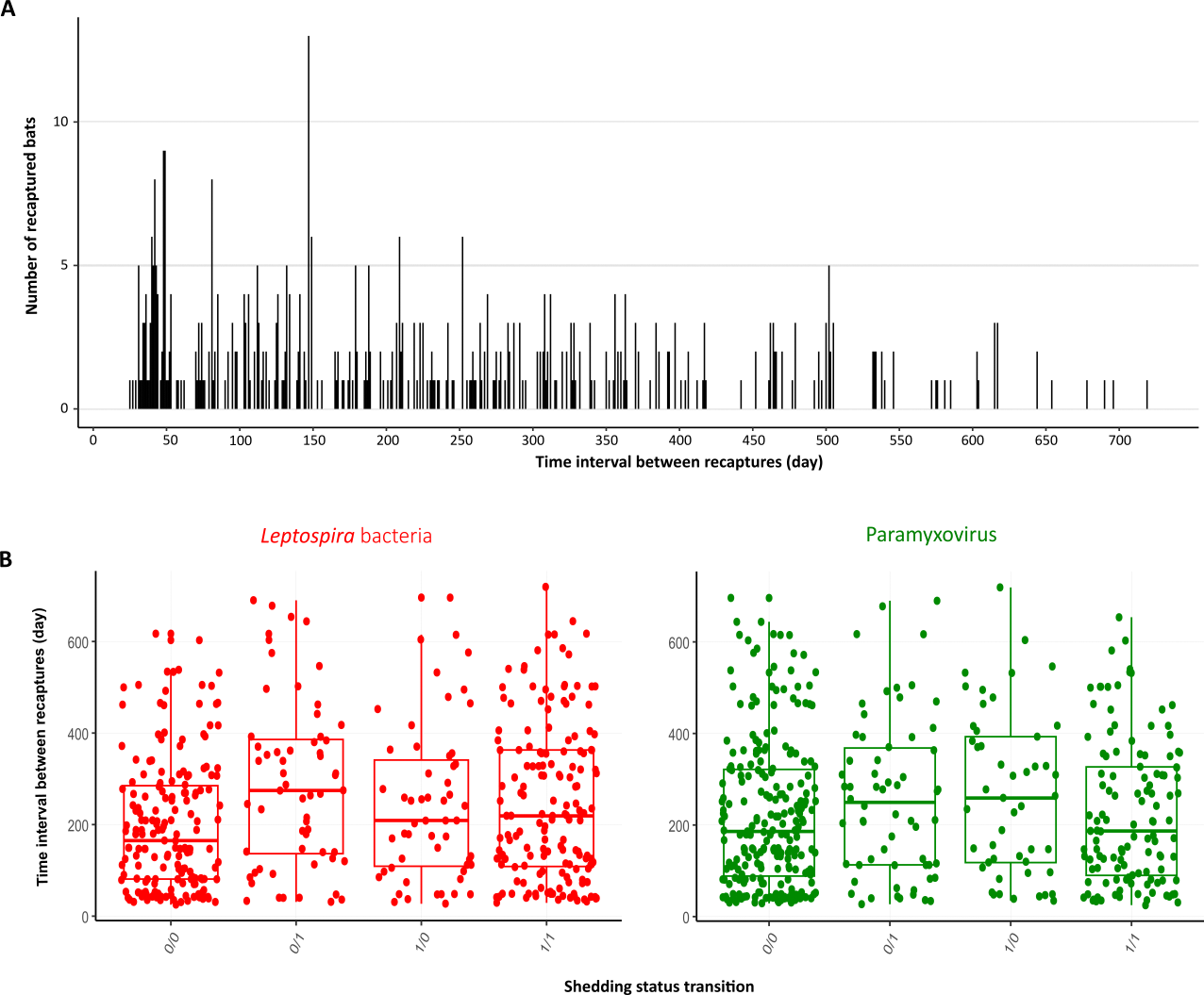
**

**S3 Fig. Details of recaptured *M. francoismoutoui* bats.** (A) Distribution of time interval between recaptures. (B) Variation of time intervals across the four categories of shedding status transitions, for *Leptospira* and paramyxovirus. Shedding status is coded with 0 for non-shedding and 1 for shedding bats.
