# Supplementary material for: Role of individual and population heterogeneity in shaping dynamics of multi-pathogen shedding in an island endemic bat
Source: PLoS Pathog. 2025 Jul 11;21(7):e1013334. doi: 10.1371/journal.ppat.1013334 (PMC12273948; doi:10.1371/journal.ppat.1013334)
Supplement: S4 Fig — The time interval (in days) between two recaptures is indicated above the arrow. (DOCX) [file ppat.1013334.s010.docx]

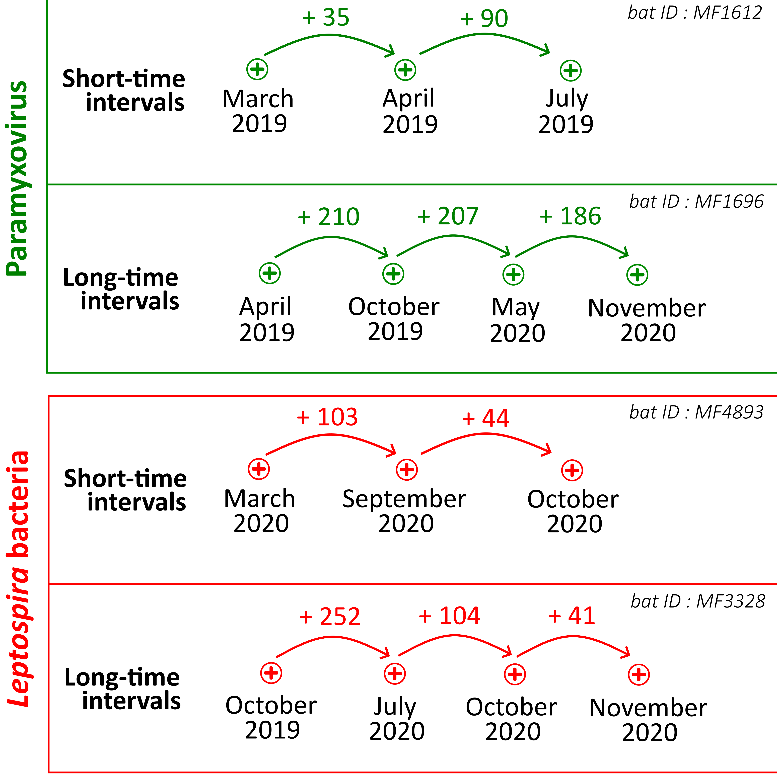


**S4 Fig. Examples of recaptured bats with always-positive status for paramyxovirus and *Leptospira*.** The time interval (in days) between two recaptures is indicated above the arrow.
