# Supplementary material for: Role of individual and population heterogeneity in shaping dynamics of multi-pathogen shedding in an island endemic bat
Source: PLoS Pathog. 2025 Jul 11;21(7):e1013334. doi: 10.1371/journal.ppat.1013334 (PMC12273948; doi:10.1371/journal.ppat.1013334)
Supplement: Attachment — Submitted filename: PPATHOGENS-D-24-01674_comments.pdf [file ppat.1013334.s011.pdf]

# PLOS Pathogens

## Role of individual and population heterogeneity in shaping dynamics of multi-pathogen excretion in an island endemic bat

--Manuscript Draft--

| | |
|---|---|
| **Manuscript Number:** | PPATHOGENS-D-24-01674 |
| **Full Title:** | Role of individual and population heterogeneity in shaping dynamics of multi-pathogen excretion in an island endemic bat |
| **Short Title:** | Drivers of co-excretion dynamics in bats |
| **Article Type:** | Research Article |
| **Section/Category:** | Evolutionary Biology - Virus |
| **Keywords:** | bats; tropical island; co-excretion dynamics; ecological factors; persistent infection |
| **Abstract:** | Understanding processes driving pathogen transmission in bats is critical to prevent spillovers and emergence events. However, it requires fine-scale ecological investigations, which are rare because of the complexity and heterogeneity of bat populations. Here, based on the monitoring of 5,949 Reunion free-tailed bats (Mormopterus francoismoutoui) in 17 roosts over 24 months, we studied the simultaneous excretion of paramyxovirus and Leptospira bacteria in urine, and herpesvirus in saliva. We analysed the influence of individual and roost-level factors on co-excretion dynamics. We evidenced all year long excretion with high prevalence (37% - 87%), with low levels of temporal synchrony among roosts and no signal of density-dependent transmission. Recapture data support persistent infections for all infectious agents, alongside with clearance followed by reinfection for paramyxovirus and Leptospira. Our results suggest that sex-specific social behaviour or physiological adaptations drive herpesvirus excretion, while paramyxovirus and Leptospira excretion reactivate during reproduction. Our data also suggest the protection of juveniles by maternal antibodies, but different waning dynamics, much shorter for HSV infection, that could lead to a pathogen-dependent role of juveniles in the spread of infection. Interestingly, the excretion of one infectious agent was positively correlated to the excretion of a second one. However, the probability of excreting a third one was reduced for individuals already bi-excreting, suggesting negative interactions for cumulative infections. Connecting such spatio-temporal data with ecosystem alterations in Reunion Island could further contribute to our understanding of the links between human-mediated changes in the ecology of bats and infectious diseases emergence processes. |
| **Additional Information:** | |
| **Question** | **Response** |
| **Government Employee**<br><br>Are you or any of the contributing authors an employee of the United States government?<br><br>Manuscripts authored by one or more US Government employees are not copyrighted, but are licensed under a CC0 Public Domain Dedication, which allows unlimited distribution and reuse of the article for any lawful purpose. This is a legal requirement for US Government employees. | No - No authors are employees of the U.S. government. |

| | |
|---|---|
| This will be typeset if the manuscript is accepted for publication. | |
| **Financial Disclosure**<br><br>Enter a financial disclosure statement that describes the sources of funding for the work included in this submission. Review the submission guidelines for detailed requirements. View published research articles from *PLOS Pathogens* for specific examples.<br><br>This statement is required for submission and **will appear in the published article** if the submission is accepted. Please make sure it is accurate.<br><br>**Funded studies**<br>Enter a statement with the following details:<br>• Initials of the authors who received each award<br>• Grant numbers awarded to each author<br>• The full name of each funder<br>• URL of each funder website<br>• Did the sponsors or funders play any role in the study design, data collection and analysis, decision to publish, or preparation of the manuscript?<br><br>Did you receive funding for this work? | Yes |
| Please add funding details.<br>as follow-up to "**Financial Disclosure**<br><br>Enter a financial disclosure statement that describes the sources of funding for the work included in this submission. Review the submission guidelines for detailed requirements. View published research articles from *PLOS Pathogens* for specific examples.<br><br>This statement is required for submission and **will appear in the published article** if the submission is accepted. Please make sure it is accurate. | This research was supported by the French National Research Agency (ANR JCJC SEXIBAT, ANR-17-CE35- 0008- 01) and by the European Regional Development Funds (ERDF PO INTERREG V ECOSPIR number RE6875). |

| | |
|---|---|
| **Funded studies**<br>Enter a statement with the following details:<br>• Initials of the authors who received each award<br>• Grant numbers awarded to each author<br>• The full name of each funder<br>• URL of each funder website<br>• Did the sponsors or funders play any role in the study design, data collection and analysis, decision to publish, or preparation of the manuscript?<br><br>Did you receive funding for this work?" | |
| Please select the country of your main research funder (please select carefully as in some cases this is used in fee calculation).<br>as follow-up to "**Financial Disclosure**<br><br>Enter a financial disclosure statement that describes the sources of funding for the work included in this submission. Review the submission guidelines for detailed requirements. View published research articles from *PLOS Pathogens* for specific examples.<br><br>This statement is required for submission and **will appear in the published article** if the submission is accepted. Please make sure it is accurate.<br><br>**Funded studies**<br>Enter a statement with the following details:<br>• Initials of the authors who received each award<br>• Grant numbers awarded to each author<br>• The full name of each funder<br>• URL of each funder website<br>• Did the sponsors or funders play any role in the study design, data collection and analysis, decision to publish, or preparation of the manuscript?<br><br>Did you receive funding for this work?" | RÉUNION - RE |

| | |
|---|---|
| **Competing Interests**<br><br>On behalf of all authors, disclose any competing interests that could be perceived to bias this work.<br><br>This statement will be typeset if the manuscript is accepted for publication.<br><br>Review the instructions link below and PLOS Pathogens' competing interests policy to determine what information must be disclosed at submission. | We have no competing interests to declare. |
| **Data Availability**<br><br>Provide a **Data Availability Statement** in the box below. This statement should detail where the data used in this submission can be accessed. This statement will be typeset if the manuscript is accepted for publication.<br><br>Before publication, authors are required to make all data underlying their findings fully available, without restriction. Review our PLOS Data Policy page for detailed information on this policy. Instructions for writing your Data Availability statement can be accessed via the Instructions link below. | Metadata are archived on Zenodo (https://doi.org/10.5281/zenodo.12527877). |

*Powered by Editorial Manager® and ProduXion Manager® from Aries Systems Corporation*

Manuscript

Click here to access/download;Manuscript;AGUILLON_MANUSCRIPT_INFE

# Role of individual and population heterogeneity in shaping dynamics of multi-pathogen excretion in an island endemic bat

Samantha Aguillon[1*], Magali Turpin[1], Gildas Le Minter[1], Camille Lebarbenchon[1], Axel O. G. Hoarau[1], Céline Toty[1], Avril Duchet[1], Léa Joffrin[1#], Riana V. Ramanantsalama[1$], Pablo Tortosa[1], Patrick Mavingui[1] & Muriel Dietrich[1*]

[1]UMR PIMIT (Processus Infectieux en Milieu Insulaire Tropical), Université de la Réunion / INSERM1187 / CNRS9192 / IRD249, 97490 Sainte-Clotilde, Reunion Island

[#]Current address: Evolutionnary Ecology Group, Department of Biology, University of Antwerp, Universiteitsplein 1, 2610 Antwerp, Belgium

[$]Current address: Behavioral Ecology & Sociobiology Unit, German Primate Center, Kellnerweg 4, 37077 Göttingen, Germany

*Samantha Aguillon - UMR PIMIT, Sainte-Clotilde, Reunion Island.

**Email:** samantha.aguillon@univ-reunion.fr

*Muriel Dietrich - UMR PIMIT, Sainte-Clotilde, Reunion Island.

**Email:** muriel.dietrich@ird.fr

## Author Contributions:

Samantha Aguillon: Conceptualization (equal); formal analysis (lead); investigation (equal); visualization (lead); writing – original draft (lead); writing – review and editing (equal). Magali Turpin: Investigation(equal); writing – review and editing (equal); Gildas Le Minter: Investigation(equal); writing – review and editing (equal). Camille Lebarbenchon: Investigation (equal); writing – review and editing (equal). Axel O. G. Hoarau: Investigation (equal); writing – review and editing (equal). Céline Toty:

Investigation (equal); writing – review and editing (equal). Avril Duchet: Investigation (equal);writing – review and editing (equal). Léa Joffrin: Investigation (equal);writing – review and editing (equal). Riana V. Ramanantsalama: Investigation (equal); writing – review and editing (equal). Pablo Tortosa: Funding acquisition (equal); investigation (equal); writing – review and editing (equal). Patrick Mavingui: Supervision (equal); writing – review and editing (equal). Muriel Dietrich: Conceptualization (lead); formal analysis (equal); funding acquisition (equal); investigation (lead); supervision (equal); validation (lead); writing – original draft (equal); writing – review and editing (equal).

## Competing Interest Statement:

We have no competing interests to declare.


## Keywords

bats; tropical island; co-excretion dynamics; ecological factors; persistent infection


## Short title

Drivers of co-excretion dynamics in bats

## Abstract

Understanding processes driving pathogen transmission in bats is critical to prevent spillovers and emergence events. However, it requires fine-scale ecological investigations, which are rare because of the complexity and heterogeneity of bat populations. Here, based on the monitoring of 5,949 Reunion free-tailed bats (*Mormopterus francoismoutoui*) in 17 roosts over 24 months, we studied the simultaneous excretion of paramyxovirus and *Leptospira* bacteria in urine, and herpesvirus in saliva. We analysed the influence of individual and roost-level factors on co-excretion dynamics. We evidenced all year long excretion with high prevalence (37% - 87%), with low levels of temporal synchrony among roosts and no signal of density-dependent transmission. Recapture data support persistent infections for all infectious agents, alongside with clearance followed by reinfection for paramyxovirus and *Leptospira*. Our results suggest that sex-specific social behaviour or physiological adaptations drive herpesvirus excretion, while paramyxovirus and *Leptospira* excretion reactivate during reproduction. Our data also suggest the protection of juveniles by maternal antibodies, but different waning dynamics, much shorter for HSV infection, that could lead to a pathogen-dependent role of juveniles in the spread of infection. Interestingly, the excretion of one infectious agent was positively correlated to the excretion of a second one. However, the probability of excreting a third one was reduced for individuals already bi-excreting, suggesting negative interactions for cumulative infections. Connecting such spatio-temporal data with ecosystem alterations in Reunion Island could further contribute to our understanding of the links between human-mediated changes in the ecology of bats and infectious diseases emergence processes.

## Author summary

Understanding risks of bat-borne pathogen spillover is challenging because of the difficulty in studying excretion dynamics in wild bat populations. Here, we used an original island-endemic model to build up a fine-scale spatio-temporal analysis of the excretion of two viruses (paramyxovirus and herpesvirus) and a bacterium (*Leptospira*) at both population- (roost) and individual- (through recaptured bats) levels. Excretion patterns are specific to each infectious agent and driven by the age and the reproduction of both females and males. Results also suggest that a combination of viral persistence and clearance, as well as interactions between infectious agents, are important within-host processes that contribute to the transmission of infections in bat populations. This information is crucial to better predict shedding pulses and prevent potential future spillovers.

## Introduction

Recent human outbreaks, such as those caused by the SARS-CoV-2 virus, have highlighted the importance of understanding processes driving the transmission of infectious agents within bat populations [1]. Indeed, a direct link between infection dynamics in reservoir bats and zoonotic spillovers has been pointed out for several viral human diseases [2–4]. Thus, identifying the ecological drivers of pathogen transmission within bat populations, at both within- and between-host levels, is a key element for predicting spillover events [5, 6]. Unfortunately, ecological studies investigating infection dynamics in bats often lack ~~of~~ robust spatio-temporal datasets [7]. This gap mainly results from logistical constraints due to the elusive and roaming nature of bats that make these animals difficult to study in long-term periods, and limits the breadth of data acquisition. Moreover, as gregarious mammals, bats often display metapopulation dynamics [8]. Such spatio-temporal heterogeneity is characterized by periodically interacting spatial discrete patches, with various sex and age-structure, roost size and function [9, 10], which adds complexity to disentangle individual and populational drivers of infection dynamics in bats [11–13].

With molecular tools becoming increasingly accessible for the study of infectious agents, it is now recognized that co-infections are common in bats [14–18]. However, most of the previous longitudinal studies on bats have focused on a single infectious agent, or different variants within the same viral family (e.g. [19–22]). Thus, our knowledge on spatio-temporal co-infection dynamics with different groups of infectious agents (DNA/RNA viruses, bacteria, fungi) remains limited [23–25]. Theory predicts that co-infections can have major consequences on within-host infection dynamics because of potential interactions that might be antagonistic or mutualistic [17, 26]. For example, the infection of field voles with the bacterium *Anaplasma phagocytophilum* increased by five times the susceptibility to cowpox virus [27]. Examining the spatio-temporal patterns of simultaneous excretion of infectious agents within bat roosts (co-circulation) and individual hosts (co-infection) is likely to provide much-needed further insights into underlying processes driving infection dynamics [21].

Island are widely considered as relevant model systems in ecology and evolutionary biology studies [28, 29], including those focusing on emerging diseases (e.g. [30, 31]). Islands are well-known ecosystems hosting limited species number and high levels of endemism [32]. Due to their ability to flight over water, bats are often the only native mammals in remote oceanic islands and among the 1,456 recognized bat species, 60% live in island and 25% are even endemic [33]. The clear boundaries and often reduced surface of islands can help sample island endemic bat populations at a fine scale, which is crucial to make reliable inferences about spatial and temporal infection dynamics [34]. Moreover, the reduced number of bat species on islands may also simplify ecological interactions, by minimizing interspecies contacts, and by sampling monospecific bat roosts, which can contribute to a more in-depth understanding of infection dynamics at the population level. Due to the reduced species richness typical of small territories, insular ecosystems may also lack predators or competitors and offer empty ecological niches, which can favor the spread of island endemic bats and the expansion of their population size. This is especially true for bat families with highly gregarious behavior such as free-tailed bats (Molossidae), for which roosts can host hundreds of thousands of individuals [35, 36]. High bat densities favor the sampling of large numbers of individuals, a prerequisite for epidemiological studies. Island endemic bats, with such an abundant, simplified, and well-defined system, represent a rare opportunity to collect extensive spatio-temporal data to capture a comprehensive picture of subtle mechanisms underlying infection dynamics in bats [37].

The Reunion free-tailed bat (*Mormopterus francoismoutoui*; [38]) is a small tropical insectivorous bat endemic to Reunion Island. This volcanic territory is located in the southwestern Indian Ocean and has emerged about 3 million years ago [39]. Although small in size (2,512 km²), Reunion Island is shaped by a mountainous landscape and has undergone significant landscape changes following 350 years of human colonization associated with intensive urbanization and agriculture [40]. Reunion free-tailed bats are broadly distributed on the island and monospecific roosts occur in a diversity of natural habitats, such as caves and cliffs. These mollosid bats have also adapted to urbanization

and use numerous anthropogenic roosts such as buildings and bridges, hosting from a few hundred to more than 100,000 individuals [9, 38]. A recent longitudinal monitoring of several roosts revealed highly dynamic roosting behaviors, with roosts of various sizes, sex-ratio, and functions (female-biased maternities, mixed colonies or male-biased roosts) [9]. Specifically, large female aggregations (up to 50,000 pregnant females) are observed synchronously every year in a few roosts, before parturition in austral summer (December), which coincides with a female-biased sex-ratio and a spatial sexual segregation during this period of the year [9, 41]. Moreover, the end of austral summer (May) is characterized by a decrease in roost size and a change in sex-ratio in the studied roosts (from female- to male-biased), suggesting important seasonal sex-specific movements within the island, which is corroborated by the absence of spatial genetic structure in this metapopulation [42].

Reunion free-tailed bats are natural hosts for several infectious agents [19, 23, 43], including paramyxovirus and *Leptospira* bacteria [41]. Previous non-invasive sampling of bat faeces and urine underneath roosts highlights that shedding pulses occurred during the austral summer, followed by a sustained transmission during winter [23, 41, 43]. However, these studies were based on a reduced number of samples, which were not associated to information on bat individuals (because no bat capture was performed), precluding direct insights into the role of individual factors (e.g. sex, age, reproductive status, body condition) shaping these infection dynamics. Moreover, investigations were respectively performed on a single [41] and two maternity roosts [23], and the role of population-level factors (e.g. roost size, sex-ratio) thus remains poorly understood. Here, we used data from a fine-scale spatio-temporal monitoring and sampling of *M. francoismoutoui*, based on the study of 17 roosts all over Reunion Island during two consecutive years. We measured the simultaneous excretion (nucleic acid detection) of three infectious agents in each individual bat, focusing on paramyxovirus (viral RNA) and *Leptospira* (bacteria DNA) both shed in urine, and herpesvirus (viral DNA) in the saliva. Specifically, we first aimed at analysing spatio-temporal trends in excretion prevalence and ~~at testing for~~ roost synchronicity. Then, we evaluated whether individual

(sex, age, reproductive status) and roost-level (roost size, sex-ratio and level of sexual spatial segregation) variables explain prevalence of single infectious agents, co-excretion patterns, and the probability of changing excretion status (within-host dynamics).

# Results

**Spatio-temporal dynamics of excretion prevalence.**

Between January 2019 and December 2020 (24 months), we captured 5,949 Reunion free-tailed bats in 17 roosts all over Reunion Island (Fig. 1), including 526 recaptures (up to four recaptures were recorded for the same individual). Excretion data were obtained from 5,518 urine samples for paramyxoviruses (PMV) and *Leptospira* bacteria (LEPTO) and from 3,981 saliva samples for herpesviruses (HSV), including 3,784 individuals for which all three infectious agents were tested. Over the entire study period, we measured a high global prevalence with 36.63% ± 1.27% of bats shedding PMV, 46.48% ± 1.32% shedding LEPTO and 87.14% ± 1.04% shedding HSV (Table 1). We used generalized additive mixed models (GAMM, *SI Appendix,* Tables S1 and S2) to characterize variation in prevalence over space and time. Results revealed no difference in prevalence between the two consecutive years, except for HSV (M3: $\chi^2_{0.8} = 4.64$, $P < 0.01$). ~~Globally,~~ we found statistical evidence of intra-annual variations for PMV and HSV prevalence (M1: $\chi^2_{6.7} = 16.30$, $P = 0.04$; M3: $\chi^2_{8.2} = 40.06$, $P < 0.001$), but when investigating data in adults only, similar temporal patterns were observed for all three infections, with a higher prevalence in October-December and a decrease from March to June (Fig. 2A).

**Table 1. Details on the sampled roosts of *M. francoismoutoui* and the number of tested and positive samples.**

| Roost | Habitat | No. of sampling periods | Roost size estimation (min and max when available) | No. of urine tested | No. of PMV positive (%) | No. of LEPTO positive (%) | No. of saliva tested | No. of HSV positive (%) |
|---|---|---|---|---|---|---|---|---|
| AOM | Building | 1 | 2560 | 52 | 6 (11.5) | 10 (19.2) | 0 | no data |
| EGI | Building | 3 | no data | 149 | 56 (37.6) | 79 (53.0) | 89 | 82 (92.1) |
| ESA | Bridge | 15 | 570 - 3200 | 634 (PMV)<br>635 (LEPTO) | 254 (40.0) | 360 (56.7) | 452 | 406 (89.9) |
| MON | Bridge | 15 | 210 - 1150 | 488 | 144 (28.4) | 268 (54.9) | 419 | 355 (84.5) |
| PBV | Cliff | 1 | 300 | 11 | 4 (36.4) | 8 (72.7) | 0 | no data |
| PSR | Building | 15 | no data | 512 (PMV)<br>510 (LEPTO) | 255 (49.8) | 305 (59.8) | 396 | 387 (97.8) |
| PSR-SUD | Building | 1 | no data | 51 | 9 (17.6) | 14 (27.4) | 0 | no data |
| RBL | Bridge | 15 | 350 - 1300 | 658 | 238 (36.2) | 235 (35.7) | 425 | 369 (86.8) |
| RES | Bridge | 8 | 35 - 410 | 223 | 91 (41.0) | 111 (49.8) | 212 | 179 (84.4) |
| RPQ | Bridge | 15 | 150 - 3000 | 535 (PMV)<br>536 (LEPTO) | 229 (41.9) | 287 (53.4) | 416 | 386 (92.8) |
| STJ | Building | 1 | 200 | 34 | 14 (41.2) | 10 (29.4) | 0 | no data |
| STM | Bridge | 12 | 27 - 350 | 364 | 145 (39.9) | 166 (45.6) | 281 | 267 (95.0) |
| TBA* | Cave | 11 | 0 - 100,000 | 500 | 140 (28.0) | 168 (33.6) | 330 | 217 (65.8) |
| TGI* | Building | 15 | 75 - 1270 | 496 | 167 (33.7) | 229 (46.1) | 432 | 356 (82.4) |
| TM5 | Bridge | 1 | 200 | 28 | 0 | 4 (14.3) | 0 | no data |
| TRI | Bridge | 4 | 153 - 251 | 125 | 45 (36.0) | 86 (68.8) | 112 | 104 (92.9) |
| VSP | Bridge | 15 | 15 - 6000 | 658 | 224 (34.0) | 225 (34.2) | 417 | 361 (86.6) |
| Total | / | / | / | 5,518 | 2,021 (36.6) | 2,565 (46.5) | 3,981 | 3,469 (87.1) |

Excretion of the three infectious agents was detected in all tested roosts, except PMV in the TM5 roost (temporary roost exclusively composed of juveniles; see [9]). Globally, no variation of PMV prevalence was observed among roosts, although we detected a significant difference for HSV and LEPTO (M2: $\chi^2_{8.2} = 96.73$, $P < 0.001$; M3: $\chi^2_{4.4} = 9.61$, $P = 0.02$). Finally, a low level of temporal synchrony in prevalence was detected among roosts, as only 11.1 % of pairwise comparisons were significant (*SI Appendix,* Fig. S1), and synchrony did not increase among nearby roosts (PMV: $r^2 = -0.11$; LEPTO: $r^2 = 0.42$; HSV: $r^2 = -0.06$; all $P > 0.05$).

**Roost-level drivers of excretion.**

Roost size varied from 15 to 100,000 individuals but was not correlated with the prevalence of PMV and LEPTO (M1: $z = 0.62$; M2: $z = 1.41$; all $P > 0.05$), while HSV

prevalence slightly decreased in larger roosts (M3: z = -2.92, $P < 0.01$). However, this negative correlation was driven by the low HSV prevalence in juveniles (the period when roost sizes were the highest), and disappeared when juveniles were removed from the model (M3 without juveniles: z = -0.99, $P = 0.32$). Moreover, prevalence was not correlated with the sex-ratio within roosts (M1: z = 0.01; M2: z = -1.05; M3: z = -1.81; all $P > 0.05$) nor with sexual segregation levels of adults among roosts (PMV: $r^2 = 0.39$; LEPTO: $r^2 = 0.43$; HSV: $r^2 = 0.51$, all $P > 0.05$).

**Individual variation of excretion.**

Models revealed that adults were systematically more frequently shedding viruses and bacteria than juveniles (M1: z = -5.82; M2: z = -6.13; M3: z = -11.56, all $P < 0.001$). Mean prevalence of excretion in adults was 43.00 ± 1.42% for PMV, 52.85 ± 1.43% for LEPTO and 96.20 ± 0.65% for HSV. At the time when juveniles started to fly (January-February; [9]), excretion prevalence in juveniles was either null (PMV) or low (LEPTO: 12.03 ± 4.11% and HSV: 15.03 ± 5.66%). Then, over the following six months, HSV prevalence increased drastically in juveniles to reach 60.67 ± 7.82%, while it remained low, or only slightly increased, for PMV and LEPTO (Fig. 2A). Excretion prevalence also varied according to sex, but only in adult bats, and differently according to the targeted infectious agent. Indeed, adult males were more frequently shedding HSV than females (Tukey's post hoc test: t = -4.10, $P < 0.001$), although the opposite pattern was observed for PMV (Tukey's post hoc test, t = 4.57, $P < 0.001$).

To test the influence of reproduction on the excretion status in adults, we focused our analysis on adults during two reproductive periods: pregnancy (November-December) and mating (March-April). During pregnancy, the detection of the two urine-excreted agents, PMV and LEPTO, was higher in pregnant than in non-pregnant females (M4: $\chi_1 = 37.49$; M5: $\chi_1 = 24.99$, all $P < 0.001$, Fig. 2B). Similarly, during the mating period, reproductively active males were more frequently found shedding than non-reproductive males, but only for LEPTO (M8: $\chi_1 = 6.76$, $P < 0.01$, Fig. 2B). Higher HSV prevalence was also observed in both reproductively active females and males, but the difference was

not statistically significant, probably because of a high variability within the non-reproductive groups.

**Co-excretion dynamics.**

Among the 3,784 bats with paired samples (both urine and saliva collected), 36.89% (n = 1,396) were shedding two infectious agents and 22.46% (n = 850) were shedding three (*SI Appendix,* Fig. S2). Only 399 bats (10.54%) did not excrete any of the three screened infectious agents, with 79.2% of them being noteworthily juveniles. The excretion of one infectious agent was strongly positively correlated with the excretion of any other infection (for all combinations in M1, M2, M3, all $P < 0.001$). Indeed, when considering bats shedding one infectious agent, the probability of shedding a second infectious agent was multiplied from 1.29 to 31.9 folds, for HSV when shedding LEPTO and for PMV when shedding HSV, respectively (Fig. 3). By contrast, individuals already shedding two infectious agents were less frequently shedding the third one (M1: $z = -3.12$, $P < 0.01$; M2: $z = -2.42$, $P = 0.02$; M3: $z = -2.42$, $P = 0.02$). For example, *Leptospira* prevalence increased by 3.89 times when bats were already shedding HSV, and by 6.50 times when already shedding PMV, but only by 2.60 times when shedding both HSV and PMV (Fig. 3).

Models showed no significant intra-annual variations for bi- and tri-excretions, except for PMV-HSV excretion (M12: $\chi_{6.7} = 19.03$, $P = 0.02$), and no effect either of sex-ratio, sexual segregation or roost size. By contrast, we detected a strong influence of age and sex, with bi- and tri-excretions being more frequent in adults (M10: $z = -4.74$; M11: $z = -10.70$; M12: $z = -7.37$; M13: $z = -4.71$; all $P < 0.001$), especially in adult females (Tuckey's post hoc test, M10: $t = 4.68$, $P < 0.001$; M12: $t = 3.68$, $P < 0.01$, M13: $t = 4.30$, $P < 0.001$). Finally, pregnancy increased the probability of bi- and tri-excretions in females (M14: $\chi_1 = 19.85$; M15: $\chi_1 = 23.04$; M16: $\chi_1 = 28.81$; M17: $\chi_1 = 17.39$; all $P < 0.001$) and reproductively active males had a higher probability of shedding simultaneously LEPTO and HSV compared to non-reproductive males (M19: $\chi_1 = 8.36$; $P < 0.01$).

**Within-host dynamics of excretion.**

All captured bats were marked (by tattooing on the wing) allowing to collect excretion data at the individual level. We tested 471 and 457 samples for PMV and LEPTO respectively, including initial capture and samples from recaptured bats. We also tested 86 saliva samples from 31 recaptured bats (from 2 to 5 capture events) for the presence of HSV. All bats initially testing positive for HSV (n = 29) remained positive over time (between 25 and 405 days between recapture events), while the only two initially-negative bats were testing positive at their first recapture. Thus, within-host analyses using multinomial logistic regressions of excretion status changes were only performed on PMV and LEPTO data. Recapture intervals ranged from 25 to 719 days (*SI Appendix,* Fig. S3A), during which we defined four conversion classes: negative to negative (no conversion), negative to positive, positive to negative, and positive to positive (no conversion). For many recaptured animals, bats kept the same excretion status over time. Indeed, between 50.74% (PMV) and 40.04% (LEPTO) of recaptured bats remained negative or, to a lesser extent (PMV: 25.69%, LEPTO: 34.79%) positive. Interestingly, when looking at short windows of time (from 1 to 6 month-intervals), a major proportion (about 75%) of PMV or LEPTO-positive bats remained positive (Fig. 4A). Some bat individuals remained positive across all their recapture events (Fig. 4B).

The probability of the four excretion status changes was dependent on the time interval for LEPTO (M23: $\chi^2_3 = 17.60$, $P < 0.001$, Fig. 4C and *SI Appendix,* Fig. S3B), but not for PMV (M22, $\chi^2_3 = 5.11$, $P = 0.16$, *SI Appendix,* Fig. S3C). Indeed, Figure 4 showed that LEPTO-negative bats had a higher probability of remaining negative over short periods of time, although the probability of becoming positive increased with time. However, when individuals were shedding LEPTO at the time of their first capture, the probability of becoming negative was not dependent on the time. In contrast, the probability of being still shedding LEPTO did increase with time. We also found a significant effect of changing reproductive status on the probability of changing both PMV and LEPTO excretion status (M22: $\chi^2_9 = 32.45$, $p < 0.001$; M23: $\chi^2_9 = 20.11$, $P = 0.02$, *SI Appendix,* Fig. S4). Indeed, bats that remained non-reproductively active over recapture events (reproduction: 0/0), also mainly remained LEPTO- and PMV-negative (conversion: 0/0). Similarly, most bats

successively captured when reproductively active (reproduction: 1/1) remained LEPTO- and PMV-positive (conversion: 1/1). Most bats becoming positive across recaptures also became reproductively active, especially for PMV (20.97% for PMV and 11.67% for LEPTO).

## Discussion

Using an original island-endemic bat model, we demonstrated the widespread and all-year-long co-circulation of three viral and bacterial infections in Reunion free-tailed bat populations, which confirms the circulation of PMV and LEPTO [23, 41] and describes for the first time HSV infection in this bat species. We revealed high global prevalence of PMV (37%) and LEPTO (46%), and extremely high prevalence of HSV (87%). This is coherent with the high prevalence of HSV generally found in other bat species, as recently reported in the Mexican free-tailed bat *Tadarida brasiliensis* [44] and in the common vampire bat *Desmodus rotundus* [12, 45]. High infection prevalence in Reunion free-tailed bats could be explained by a strong gregarious behaviour of these bats, which can aggregate up to 900 individuals per m² [46]. The proximity of individuals within high-density roosts can facilitate transmission through bat fluids (saliva, urine), in which case, roost size is often assumed to positively correlate with prevalence [4, 47, 48]. However, we did not find such a correlation, suggesting that density-dependent transmission may not play a major role in the circulation of paramyxovirus, *Leptospira* and herpesvirus in Reunion free-tailed bats. However, we cannot exclude that if prevalence is biased towards certain groups of individuals that aggregate in high density within area, then transmission is likely to be related to the density of these high-risk bat groups, rather than the overall roost size measured in our study [49]. Interestingly, we did not find evidence of spatial synchrony between roosts, despite probably frequent intra- and inter-seasonal movements within this bat metapopulation [9, 42]. Thus, transient epidemics, passing as waves of bat-to-bat transmission between roosts, are probably not the main mechanism involved in maintenance [4, 11, 50] and the probability of excretion seems therefore more related to individual factors than population-level characteristics.

Chronic or episodic shedding from persistently infected bats can explain high prevalence across roosts and time. Indeed, our results shows that nearly all (96%) of recaptured bats, initially HSV-positive, were still shedding HSV in saliva over recaptures, supporting lifelong HSV infection in Reunion free-tailed bats. HSV are known to establish latent infection in humans [51] and rodents [52], and the same mechanism is also suspected in bats [53, 54]. Interestingly, in *D. rotundus* infected with a betaherpesvirus, the length of the active periods (when shedding occurs) appears to be longer than the latency state (when shedding resumes) [54], which is coherent with our recapture data showing almost always HSV-positive bats over recaptures. For PMV and LEPTO, we showed that viral RNA and bacterial DNA were present in urine for up to 3 months after their initial detection in a large proportion of recaptured bats (75%). Together with examples of bats remaining positive over multiple recapture events, this supports that persistent infection can also explain PMV and LEPTO temporal dynamics. However, we also evidenced positive to negative excretion switches for PMV and LEPTO, suggesting that cycles of clearance followed by reinfection also occur in the bat population. Persistent LEPTO infection in bats is not surprising as this bacterial infection is often considered chronic in other reservoir hosts, bacteria forming protective biofilms in renal tubules [55]. Experimental infections have shown that *Leptospira interrogans* can stably colonize the kidneys of rats and mice over their lifespan, with intermittent excretion of the bacteria that can last for at least 3 months [56, 57]. Our data show that when individuals are shedding LEPTO at the first capture, the probability of becoming negative is not dependent on the time, which is in keeping with an intermittent excretion. On contrary, mechanisms underlying within-host PMV dynamics are still debated [5, 58]. Several field and lab evidence support that henipavirus, for example, can persist within bat individuals with long-term continuous or intermittent excretion [11, 37, 59–62]. A modelling study also suggests that latent henipavirus infections can occur alongside with occasional viral clearance, followed by temporary immunity and reinfection [58]. This same study supports that bats are much more likely to develop a latent infection than clearing it. Finally, it has been hypothesized that a small portion of super-long-shedder

individuals with a long infectious period could maintain Hendra virus in bat populations [63]. Distinguishing between these different infection processes is complex. Finally, since because we only measured the presence of viral and bacterial nucleic acids in our study, an important step will be to assess the infectiousness of excreted samples to finely validate the role of long-term continuous and intermittent excretion in transmission [4].

In persistent infections, the reactivation process is often linked to several stressors that may affect host immunity [64, 65]. Reproduction, by imposing immunological challenges, is recognized as a major stressor that could lead to the reactivation of viral and bacterial infections [66]. Such processes are expected to be particularly pronounced in female mammals, for whom reproduction requires large and prolonged energetic investments [67, 68]. We showed that pregnancy increased the probability of PMV and LEPTO (but not HSV) excretions, as well as bi- and tri-excretions in Reunion free-tailed bats. Variable patterns between infectious agents are coherent with the increased seroprevalence observed in reproductively active females of *P. scapulatus* infected with Hendra virus [69], and the absence of such correlation for HSV-infected *Rousettus aegyptiacus* and *Miniopterus natalensis* [70]. Moreover, recapture data showed that most bats becoming PMV and LEPTO positive across recaptures were also those becoming reproductively active. We thus hypothesize that both PMV and LEPTO excretions are reactivated during female's pregnancy in Reunion free-tailed bats. In humans, such process has been described for the Varicella Zoster Virus, for which reactivation occurs commonly during the third trimester of pregnancy [71]. Reactivation during bat pregnancy could explain the enhanced prevalence observed during the pregnancy period (October to December), a pattern reported in other field studies [19, 20, 23, 41, 70, 72]. Such reactivation could also lead to horizontal transmission to non-immune bats. However, we cannot exclude that immigration of infected bats also has a role in transmission during late pregnancy, as massive aggregations of bats occur at this time of the year [9]. Our study therefore supports the role of a trade-off between pregnancy and immunity. Some studies have hypothesized that such process, beside conferring

protection to newborns through the transfer of protective maternal antibodies, could benefit to the mothers through a boost of immunity [71].

Although less documented, male reproduction trade-offs have been suggested in bats, with higher Hendra seroprevalence observed in *Pteropus scapulatus* males during the mating season [69]. Our study revealed that, during the mating period, reproductively active males had a higher probability of shedding LEPTO and LEPTO-HSV, as compared to non-reproductive males. By contrast, no difference was observed for PMV excretion. This suggests that trade-offs between reproduction and immunity in males could drive excretion dynamics, but that differences exist depending on the considered infectious agent. Such pattern could be mediated by reduced immune function during spermatogenesis, a period of high energy demand and elevated testosterone [73, 74]. As well, reproductively active males of *Myotis daubentonii* have lower cellular immunity than non-reproductive individuals [75].

Previous studies have shown that environmental stressors could be critical to HSV transmission in wildlife [76]. HSV was, for example, detected in the blood of big brown bats (*Eptesicus fuscus*) arousing from hibernation, but not in non-hibernating individuals, suggesting a reactivation of the virus resulting from the stress associated with arousal [53]. Accordingly, our results showed a slight increase of HSV prevalence in adult bats at the end of winter (July to November). However, Reunion free-tailed bats are tropical, and we thus suspect that no strict hibernation occurs [77], supported by observations of bat activity all year long [9]. The HSV increase in winter might alternatively result from a misclassification of juveniles in adults, because differentiating those age classes based on the epiphysis fusion is challenging at this period. Since juveniles are less infected than adults, we might have artificially lowered the prevalence in adults.

Behavioral and physiological traits are two mechanisms that can produce sex-biased prevalence in several wildlife species [78, 79]. Here, males were more frequently shedding HSV than females, as previously reported in rodents [52] or for other DNA bat viruses such as adenoviruses [80]. Studies on HSV prevalence in bats are limited and report contrasting results, with no sex difference in *D. rotundus* [12], while Sjodin et al.,

[81] suggest that either males or females can be more infected, depending of the herpesvirus strain. Male-biased infection may arise from sex-specific social behaviour like dominance or higher aggressiveness (physical injuries, i.e. bite) [82, 83], that could enhance transmission in males [52, 84]. However, this hypothesis is not well-supported in Reunion free-tailed bats, since increased transmission among aggressive males should lead to a higher prevalence during the mating season, which was not observed. Sex hormones can also strongly affect host susceptibility, through the modulation of immunity [85]. Testosterone may suppress immune responses in males, increasing susceptibility to pathogen infection [86]. The inclusion of hormone and immunity measures should thus be considered in future infection studies to test this hypothesis in bats.

Maternal transmission of antibodies conferring specific protection to juveniles is a well-known process in mammals, and has often been suggested and demonstrated in bats [2, 60, 69, 72, 87–89]. The level of maternally transferred antibodies to juveniles is thought to reflect the amount of circulating antibodies in females, and could thus positively correlate with infection prevalence [90]. Here, although the prevalence in adult females was much higher for HSV than for PMV and LEPTO, up to 20% (in 2019) of juveniles were HSV-positives at the start of the juvenile period (January), while almost all juveniles were non-infected by PMV (with an intermediate pattern observed for LEPTO). This suggests that despite a wider excretion of HSV in the bat population, initial levels of maternal antibodies could be lower for HSV, compared to PMV and LEPTO. If the waning of maternal antibodies in juveniles depends on the initial levels provided by mothers, this can explain the distinct excretion dynamics later observed in juveniles. Indeed, although most juveniles remained PMV and LEPTO negative during the first six months of their life, HSV excretion rapidly increased to reach 80% in July 2020. Contrary to HSV, the protection against PMV and LEPTO seems therefore quasi-total (although lower for LEPTO) and longer (> six months) for these two infections, suggesting different strategies of maternal immunity in bats. Apparent short-term maternal immunity has been proposed in Egyptian fruit bats (*Rousettus aegyptiacus*) infected with Marburg virus, for which juveniles'

infection increases by 5-fold within 3 months [2], while a longer protection of about 6-7 months has been reported from field and modelling studies on Lagos bat virus and henipaviruses [37, 87, 91]. Finally, the rapid and steady HSV infection in juveniles coincides with their initial dispersal all over the island [9] and could thus play a major role in the spatial spread of this virus [2, 23, 69, 72]. By contrast, the spread of PMV and LEPTO by juveniles is probably lower (or occurs later in the year), as juveniles remain poorly infected at least during their first six months of life, highlighting that the role of juveniles in infection dynamics is pathogen-dependent [63].

Our investigation targeting multiple simultaneous infections revealed a high co-circulation of viruses and bacteria within the bat population, with 59% of bats shedding at least two infectious agents. Our results also showed that bats shedding one virus or bacteria had significantly more chance of shedding another one. This corroborates previous results suggesting positive interactions that could facilitate the excretion of paramyxo- [21, 41] and corona-viruses [16, 25]. In the study of Davy et al. [16], the stress of fungal infection by *Pseudogymnoascus destructans* led to a reactivation of coronavirus replication in the little brown bat *Myotis lucifugus*, and co-infection even increased the severity of White Nose Syndrome. Interestingly, in our study, when bats were already shedding two infectious agents, they had less probability of shedding the third one, suggesting that negative interactions can also occur when accumulating infections. Interactions between infectious agents could modulate host susceptibility with contrasting effects (antagonistic and mutualistic) depending on the community of infectious agents considered [27]. In a previous investigation on Reunion free-tailed bats we indeed highlighted that coinfection between Paramyxovirus, Coronavirus and Astrovirus was a very rare event (1% of tested samples), without interaction between viruses [23]. These studies illustrate how considering simultaneously multiples infections, although complex, is important to better understand drivers of infection in bats [17, 92, 93].

This study sheds light on the processes driving the spatio-temporal excretion dynamics of RNA and DNA viruses as well as bacteria in bats. In addition to suggested

latent herpesvirus infections, our results suggest that bats commonly host persistent paramyxovirus and *Leptospira* infections, with reproduction-related episodic excretion, in both females and males. Surprisingly, transient epidemics and density-dependent transmission do not appear as major drivers of these infections. Moreover, pathogen-dependent waning immunity dynamics likely modulates the role of juveniles in the spatial spread of infections. Testing these hypotheses requires serological data and spatially explicit and mechanistic modelling studies [94]. Disruption of balanced host-pathogen relationships due to stressors, such as habitat loss or co-infections, may increase pathogen excretion, and enhance the likelihood of spillovers [3]. Further studies connecting multi-infection dynamics to the growing urbanization process that faces Reunion free-tailed bats will help in understanding how human-mediated changes in the ecology of bats may affect natural excretion dynamics and in turn the probability of spillovers [95].

## Materials and Methods

### Study sites and field sampling

We monitored and sampled 17 roosts (coded with a 3-letter code) from January 2019 to December 2020 (24 months), all over Reunion Island (Fig. 1). Among the 17 studied roosts, 12 were monitored across a maximum of 15 successive "sampling periods" occurring every 3–8 weeks, each sampling period lasting for 3–4 weeks to be able to sample multiple roosts (Table 1 and Table S3). An opportunistic sampling was performed for five additional roosts, which were thus monitored only once during the studied period. To investigate within-host dynamics, we used data from recaptured bats, and thus also included previously collected samples from 2018 (referred as the first capture samples).

Bat monitoring includes bat capture, biological sampling and roost size estimation and is fully described in Aguillon et al. [9]. Captures were carried out using harp traps (Faunatech Ausbat) and Japanese monofilament mist nets (Ecotone) during dusk emergence, with a maximum of 60 individuals captured per night. Bats were hydrated immediately after the capture and kept individually in a clean individual bag hang close

to a warm water bottle. For each bat, we measured the forearm length and mass, and determined the sex, age, and reproductive status. Age was established by examining the epiphysis fusion in finger articulations that are not welded for juveniles which was easily identifiable until July, while some juveniles might have been classified as adults in the following months. Active reproductive status was recorded when females were pregnant and lactating, and in males when they had large testes (see [9] for more details). A sterile swab (Puritan Medical Products, USA) was carefully inserted in the corner of the lips to sample saliva and then placed in 250uL of MEM (Minimum Essential Medium Eagle). Using a pipette and a sterile tip, urine droplets were collected in the waterproof bag or directly at the urethral opening when handling bats, and then placed in 50 µL of MEM. All samples were stored in a cooled box in the field before being transferred at -80°C at the laboratory the same night. Finally, each bat was tattooed on the right propatagium with an individual alphanumeric code [96] and released at the capture site.

Bats were handled using personal protective equipment regularly changed and all the equipment was disinfected between sites (more details in [9]). Bat capture and manipulation protocols were evaluated and approved by the Minister of Higher Education, Research and Innovation (APAFIS#10140-2017030119531267), and conducted under a permit delivered by the Direction de l'Environnement, de l'Aménagement et du Logement (DEAL) of Reunion Island (DEAL/SEB/UBIO/2018-09).

**Molecular detection of infectious agents**

Urine and saliva samples were processed using the Cador Pathogen 96 Qiacube HT kit (Qiagen, Hilden, Germany), using 25 µL and 100 µL of samples, respectively, mixed with 175 µL and 100 µL of VXL buffer for the lysis step. Total nucleic acids were extracted using an automated extractor Qiacube with small modifications of the Q Protocol using 350 µL of ACB, 100 µL of AVE, and 30 µL of TopElute. A reverse transcription was then performed on urine extracts [97], followed by the screening of paramyxovirus and *Leptospira* bacteria, using a semi-nested PCR targeting the RNA polymerase gene and a real-time PCR amplifying a fragment of the 16S rDNA gene, respectively [41, 98, 99]. For paramyxovirus,

amplicons of the first PCR were diluted to 1:10, and 2 µL was then used in the semi-nested PCR.

For saliva extracts, a fragment of herpesvirus DNA polymerase was amplified using a nested PCR. During the first PCR, 2 µL of DNA was amplified in a 25 µL mixture containing 12.5 µL of GoTaq® Green Master Mix 2X (Promega, Madison, Wisconsin, United States), 2.5 µL of each primer at 10 µM DFA (5'-GAYTTYGCNAGYYTNTAYCC-3') and KG1 (5'-GTCTTGCTCACCAGNTCNACNCCYTT-3') [100]. We used the following thermal conditions: 95°C for 2 min, and 45 cycles at 94°C for 30 sec, 46°C for 1 min and 72°C for 1 min, and a final step at 72°C for 7 min. During the nested PCR, 5 µL of amplificon was amplified in a 25 µL mixture containing 12.5 µL of GoTaq® Green Master Mix 2X (Promega, Madison, Wisconsin, United States), 2.5 µL of each primer at 10 µM TGV (5'-TGTAACTCGGTGTAYGGNTTYACNGGNGT-3') and IYG (5'-CACAGAGTCCGTRTCNCCRTADAT-3') [100]. We used the following thermal conditions: 95°C for 2 min, and 35 cycles at 94°C for 30 sec, 46°C for 30 sec and 72°C for 30 sec, and a final step at 72°C for 7 min. PCR products were revealed using electrophoresis with 2% agarose gel stained with 2% GelRed (Biotium, Fremont, USA).

**Statistical analyses**

To evaluate spatio-temporal dynamics of excretion, we fit binomial generalized additive mixed models (GAMM, [101]) via restricted maximum likelihood (REML) using the *mgcv* package (Table S1). We used penalized smoother for the temporal trend variable and controlled that the effective degrees of freedom (EDF) were higher than 1 (non-linear effect). The number of basis functions (k) and smooth parameters (alpha) were adjusted in each model using *gam.check* from the *mgcv* package to avoid overfitting and check for model convergence. Temporal trend was implemented specifying a number for each sampling period starting from 1 (January-February) to 16 (November-December, Table S3). Due to the COVID-19 crisis and the resulting lockdown in France, we did not monitor roosts in April 2020, and do not present data at that time. To properly account for the temporal effect, we decided to code the May-June 2020 sampling period as the middle of

March (coded as 10) and July 2020 (coded as 13, Table S3). Final models for mono-, bi- and tri- excretions included the sampling period as smooth function, the roost size, sex-ratio, age and sex (and their interaction) as linear function, and the roost, Julian day and year as random effects. The significance of each modality of age/sex interactions was tested using a Tukey's post hoc test. For mono-excretion models, we included the two other infectious agents and the corresponding bi-excretion as a linear function (details in Tables S2 and S3). Moreover, to determine periods of significant increase/decrease in the prevalence of mono-excretions in adults, we used simplified models that only included sampling period as a smooth function, and identified periods where the 95% confidence interval (CI) of the GAM prediction's derivative did not overlap zero, using the R function *derivSimulCI* (www.gist.github.com/gavinsimpson).

To assess the effect of reproductive status on excretion, we focused on adult bats at two specific periods of the year: firstly, in adult females during the pregnancy period (November-December) and then in adult males during the mating period (March-April) [9]. We thus used a separate GLMM on a subset of data, for each sex and included reproductive status as a linear effect, keeping roost, Julian day and year as random effects (Table S1 and S2).

The influence of sexual spatial segregation patterns in adults was tested on the prevalence of mono-, bi-, and tri-excretions, using the Sexual Segregation and Aggregation Statistic (SSAS, [102]) that compares observed aggregation and segregation patterns to a random association of males and females among roosts. We calculated SSAS values for each sampling period, and tested if the SSAS was correlated to the global monthly prevalence of excretion using a Pearson correlation test.

Then, we analysed the spatial (between roosts) synchrony in prevalence using the same approach as in Meza et al. [13]. We selected seven roosts (ESA, MON, PSR, RBL, RPQ, TGI and VSP) that were all monitored during every sampling periods. We used Mantel tests (from *vegan* package) to investigate geographical distances between roosts and calculated pairwise synchrony of prevalence by sampling period for each infectious agent separately, using Spearman correlations tests (Fig. S1).

To investigate within-host excretion dynamics, we tested the probability of changing infectious agent excretion status through time, by fitting a multinomial logistic regression using the *multinom* function in the *nnet* package (103] (Table S4). Because not enough saliva samples were available to analyse the conversion of HSV excretion status, we only modelled conversion status associated with PMV and LEPTO, separately. Conversion status was used as a nominal dependent variable with four levels (negative to negative, negative to positive, positive to negative, and positive to positive), with the following covariates: recapture interval (in days), sex, and reproductive status conversion (active to active, non-active to active, active to non-active and non-active to non-active). Age classes were not included in the models because of the limited number of juveniles compared to adults ($n_{JUV}$ = 29, $n_{AD}$ = 497). This analysis used the non-changing infection, negative to negative, as a baseline outcome against which to compare other infectious status changes. To better visualize the probable continuous excretion over short time windows (<6 months), we calculated and plotted the percentage of bats remaining positive for PMV and LEPTO across different time intervals: less than 2 months, between 2 and 4 months, between 4 and 6 months.

All analyses were performed in Rstudio 1.4.1106 [104] using packages *dplyr*, *effects, emmeans*, *lme4*, *ggeffects*, *ggplot2*, *mgcv, nnet,* and *vegan*.

## Acknowledgments

We are grateful to Eco-Med Océan Indien, Biotope, the DEER of Région Réunion (Direction de l'Exploitation et de l'Entretien des Routes), the DRT of Département Réunion (Direction des Routes et des Transports of the Department Reunion), and the City hall of Salazie for their help in identifying and accessing bat roosts. We are grateful to David Wilkinson for fruitful discussions. We also thank Clara Castex, Timothée Chenin, Jérémy Dubrulle, Marie Köster, and Guillaume Verchère for their assistance in the field. This research was supported by the French National Research Agency (ANR JCJC SEXIBAT) and by the European Regional Development Funds ERDF PO INTERREG V ECOSPIR number RE6875. Samantha Aguillon and Axel Hoarau were supported by a "Contrat Doctoral de

l'Université de La Réunion" from the Minister of Higher Education, Research and Innovation and Léa Joffrin by an "Allocation Régionale de Recherche de la Région Réunion".

## Data Availability

Metadata are archived on Zenodo (https://doi.org/10.5281/zenodo.12527877). A pre-print is available on bioRxiv (https://doi.org/10.1101/2024.06.25.600581).

## References

1. Letko M, Seifert SN, Olival KJ, Plowright RK, Munster VJ. Bat-borne virus diversity, spillover and emergence. Nat. Rev. Microbiol. 2020; 18: 461–471.

2. Amman BR, Carroll SA, Reed ZD, Sealy TK, Balinandi S, Swanepoel R, et al. Seasonal pulses of Marburg virus circulation in juvenile *Rousettus aegyptiacus* bats coincide with periods of increased risk of human infection. PLOS Pathog. 2012; 8: e1002877.

3. Becker DJ, Eby P, Madden W, Peel AJ, Plowright RK. Ecological conditions predict the intensity of Hendra virus excretion over space and time from bat reservoir hosts. Ecol. Lett. 2023; 26: 23–36.

4. Plowright RK, Eby P, Hudson PJ, Smith IL, Westcott D, Bryden WL et al. Ecological dynamics of emerging bat virus spillover. Proc. R. Soc. B: Biol. Sci. 2015; 282: 20142124.

5. Plowright RK, Peel AJ, Streicker DG, Gilbert AT, McCallum H, Wood J, et al. Transmission or within-host dynamics driving pulses of zoonotic viruses in reservoir–host populations. PLOS Negl. Trop. Dis. 2016; 10.

6. Sánchez CA, Phelps KL, Frank HK, Geldenhuys M, Griffiths ME, Jones DN,et al. Advances in understanding bat infection dynamics across biological scales. Proc. R. Soc. B: Biol. Sci. 2024; 291: 20232823.

7. Becker DJ, Crowley DE, Washburne AD, Plowright RK. Temporal and spatial limitations in global surveillance for bat filoviruses and henipaviruses. Biol. Lett. 2019; 15: 20190423.

8. Calisher CH, Childs JE, Field HE, Holmes KV, Schountz T. Bats: Important reservoir hosts of emerging viruses. Clin. Microbiol. Rev. 2006; 19: 531–545.

9. Aguillon S, Le Minter G, Lebarbenchon C, Hoarau AOG, Toty C, Joffrin L, et al. A population in perpetual motion: Highly dynamic roosting behavior of a tropical island endemic bat. Ecol. Evol. 2023; 13: e9814.

10. Delpietro HA, Russo RG, Carter GG, Lord RD, Delpietro GL. Reproductive seasonality, sex ratio and philopatry in Argentina’s common vampire bats. R. Soc. Open Sci. 2017; 4: 160959.

11. Epstein JH, Anthony SJ, Islam A, A. Marm Kilpatrick A, Ali Khan S, Balkey MD, et al. Nipah virus dynamics in bats and implications for spillover to humans. Proc. Natl. Acad. Sci. U.S.A. 2020; 117: 29190–29201.

12. Griffiths ME, , Bergner LM, Broos A, Meza DK, da Silva Filipe A, Davison A, et al. Epidemiology and biology of a herpesvirus in rabies endemic vampire bat populations. Nat. Commun. 2020; 11: 5951.

13. Meza DK, Mollentze N, Broos A, Tello C, Valderrama W, Recuenco S, et al. Ecological determinants of rabies virus dynamics in vampire bats and spillover to livestock. Proc. R. Soc. B: Biol. Sci. 2022; 289: 20220860.

14. Anthony SJ, Epstein JH, Murray KA, Navarrete-Macias I, Zambrana-Torrelio CM, Solovyov A, et al. A strategy to estimate unknown viral diversity in mammals. MBio. 2013; 4: e00598-13.

15. Baker KS, K. S. Baker, Todd S, Marsh G, Fernandez-Loras A, Suu-Ire R, Wood JLN, et al. Co-circulation of diverse paramyxoviruses in an urban African fruit bat population. J. Gen. Virol. 2012; 93: 850–856.

16. Davy CM, Donaldson ME, Subudhi S, Rapin N, Warnecke L, Turner JM, et al. White-nose syndrome is associated with increased replication of a naturally persisting coronaviruses in bats. Sci. Rep. 2018; 8: 15508.

17. Hoarau AOG, Mavingui P, Lebarbenchon C. Coinfections in wildlife: Focus on a neglected aspect of infectious disease epidemiology. PLOS Pathog. 2020; 16: e1008790.

18. Wang J, Pan Y-F, Yang L-F, Yang W-H, Lv K, Luo C-M, et al. Individual bat virome analysis reveals co-infection and spillover among bats and virus zoonotic potential. Nat. Commun. 2023; 14: 4079.

19. Joffrin L, Hoarau AOG, Lagadec E, Torrontegi O, Köster M, Le Minter G, et al. Seasonality of coronavirus shedding in tropical bats. R. Soc. Open Sci. 2022; 9: 211600.

20. Mortlock M, Dietrich M, Weyer J, Paweska JT, Markotter W. Co-circulation and excretion dynamics of diverse rubula-and related viruses in Egyptian rousette bats from South Africa. Viruses. 2019; 11: 37.

21. Peel AJ, Wells K, Giles J, Boyd V, Burroughs A, Edson D, et al. Synchronous shedding of multiple bat paramyxoviruses coincides with peak periods of Hendra virus spillover. Emerg. microbes & infect. 2019; 8: 1314–1323.

22. Geldenhuys M, Ross N, Dietrich M, de Vries JL, Mortlock M, Epstein JH et al. Viral maintenance and excretion dynamics of coronaviruses within an Egyptian rousette fruit bat maternal colony: considerations for spillover. Sci. Rep. 2023; 13: 15829.

23. A. O. G. Hoarau, Köster M, Dietrich M, Le Minter G, Joffrin L, Ramanantsalama RV et al. Synchronicity of viral shedding in molossid bat maternity colonies. Epidemiol. Infect. 2023; 151: e47.

24. Lane JK, Negash Y, Randhawa N, Kebede N, Wells H, Ayalew G et al. Coronavirus and paramyxovirus shedding by bats in a cave and buildings in Ethiopia. EcoHealth. 2022; 19: 216–232.

25. A. Seltmann, Corman VM, Rasche A, Drosten C, Á Czirják G, Bernard H, et al. Seasonal fluctuations of astrovirus, but not coronavirus shedding in bats inhabiting human-modified tropical forests. EcoHealth. 2017; 14: 272–284.

26. Petney TN, Andrews RH/ Multiparasite communities in animals and humans: frequency, structure and pathogenic significance. Int. J. Parasitol. 1998; 28: 377–393.

27. Telfer S, Lambin X, Birtles R, Beldomenico P, Burthe S, Paterson S, et al. Species interactions in a parasite community drive infection risk in a wildlife population. Science. 2010; 330: 243–246.

28. Warren BH, Simberloff D, Ricklefs RE, Aguilée R, Condamine FL, Gravel D, et al. Islands as model systems in ecology and evolution: prospects fifty years after MacArthur-Wilson. Ecol. Lett. 2015; 18: 200–217.

29. Emerson BC. Evolution on oceanic islands: molecular phylogenetic approaches to understanding pattern and process. Mol. Ecol. 2002; 11: 951–966.

30. Jean K, Burnside WR, Carlson L, Smith K, Guégan J. An equilibrium theory signature in the island biogeography of human parasites and pathogens. Glob. Ecol. Biogeogr. 2016; 25: 107–116.

31. Tortosa P, Pascalis H, Guernier V, Cardinale E, Le Corre M, Goodman SM, et al. Deciphering arboviral emergence within insular ecosystems. Infect. Genet. Evol. 2012; 12: 1333–1339.

32. Kier G, Kreft H, Ming Lee T, Jetz W, Ibisch PL, Nowicki C et al. A global assessment of endemism and species richness across island and mainland regions. Proc. Natl. Acad. Sci. 2009; 106: 9322–9327.

33. Conenna I, Rocha R, Russo D, Cabeza M. Insular bats and research effort: A review of global patterns and priorities. Mam. Rev. 2017; 47: 169–182.

34. Plowright RK, Becker DJ, McCallum H, Manlove KR. Sampling to elucidate the dynamics of infections in reservoir hosts. Phil. Trans. R. Soc. B. 2019; 374: 20180336.

35. Betke M, Hirsh DE, Makris NC, McCracken GF, Procopio M, Hristov NI, et al. Thermal imaging reveals significantly smaller Brazilian free-tailed bat colonies than previously estimated. J Mammal. 2008; 89: 18–24.

36. Mcfarlane D, Rentergem G, Ruina A, Lundberg J, Christenson K. Estimating colony size of the Wrinkle-lipped bat, *Chaerephon plicatus* (Chiroptera: Molossidae) at Gomantong, Sabah, by quantitative image analysis. Acta Chiropt. 2015; 17: 171–177.

37. Peel AJ, Baker KS, Hayman DTS, Broder CC, Cunningham AA, Fooks AR, et al. Support for viral persistence in bats from age-specific serology and models of maternal immunity. Sci. Rep. 2018; 8: 3859.

38. Goodman SM, van Vuuren BJ, Ratrimomanarivo F, Probst J-M, Bowie RCK. Specific status of populations in the Mascarene Islands referred to *Mormopterus acetabulosus* (Chiroptera: Molossidae), with description of a new Species. J. Mammal. 2008 ; 89: 1316–1327.

39. Cadet T. Données récentes sur l'origine, l'âge et la structure géologique de l'île de la Réunion. *Académie de l'île de la réunion Bulletin 1969-1978.* 1980; 73–87.

40. Lagabrielle E, Rouget M, Payet K, Wistebaar N, Durieux L, Baret S et al. Identifying and mapping biodiversity processes for conservation planning in islands: A case study in Réunion Island (Western Indian Ocean). Biol. Conserv. 2009; 142: 1523–1535.

41. Dietrich M, Wilkinson DA, Benlali A, Lagadec E, Ramasindrazana B, Dellagi K, et al. Leptospira and paramyxovirus infection dynamics in a bat maternity enlightens pathogen maintenance in wildlife. Environ. Microbiol. 2015; 17: 4280–4289.

42. S. Aguillon, Castex C, Duchet A, Turpin M, Le Minter G, Lebarbenchon C, et al. Landing on a small tropical island: wide in-situ diversification of an urban-dwelling bat. Glob. Ecol. Conserv. 2024; 53: e03030.

43. L. Joffrin, Hoarau AOG, Lagadec E, Köster M, Ramanantsalama RV, Mavingui P et al. Astrovirus in Reunion free-tailed Bat (*Mormopterus francoismoutoui*). Viruses. 2021; 13: 1524.

44. Moreira Marrero L, Botto Nuñez G, Malta L, Delfraro A, Frabasile S. Ecological and conservation significance of herpesvirus infection in neotropical bats. EcoHealth. 2021; 18: 123–133.

45. Griffiths ME, Broos A, Bergner LM, Meza DK, Suarez NM, da Silva Filipe A, et al. Longitudinal deep sequencing informs vector selection and future deployment strategies for transmissible vaccines. PLOS Biol. 2022; 20: e3001580.

46. Héré L, Probst J-M, Lequette B. Contribution à l'étude des chiroptères de l'île de La Réunion. Répartition et habitats prioritaires en matière de conservartion. Université de La Réunion. Rapport de stage de Master. 2009.

47. Serra-Cobo J, López-Roig M, Seguí M, Pilar Sánchez L, Nadal J, Borrás M, et al. Ecological factors associated with European bat lyssavirus seroprevalence in Spanish bats. PLOS ONE. 2013; 8: e64467.

48. Streicker DG, Recuenco S, Valderrama W, Gomez Benavides J, Vargas I, Pacheco V, et al. Ecological and anthropogenic drivers of rabies exposure in vampire bats: implications for transmission and control. Proc. Biol. Sci. 2012; 279: 3384–3392.

49. Begon M, Bennett M, Bowers RG, French NP, Hazel SM, Turner J. A clarification of transmission terms in host-microparasite models: numbers, densities and areas. Epidemiol. Infect. 2002; 129: 147–153.

50. Laughlin AJ, Hall RJ, Taylor CM. Ecological determinants of pathogen transmission in communally roosting species. Theor. Ecol. 2019; 12: 225–235.

51. Cohen JI. Herpesvirus latency. J. Clin. Invest. 2020; 130: 3361–3369.

52. Erazo D, Pedersen AB, Gallagher K, Fenton A. Who acquires infection from whom? Estimating herpesvirus transmission rates between wild rodent host groups. Epidemics. 2021; 35: 100451.

53. Gerow CM, Rapin N, Voordouw MJ, Elliot M, Misra V, Subudhi S. Arousal from hibernation and reactivation of *Eptesicus fuscus* gammaherpesvirus ( *Ef* HV ) in big brown bats. Transbound Emerg. Dis. 2018; 66: 1054–1062.

54. Griffiths ME, Meza DK, Haydon DT, Streicker DG. Inferring the disruption of rabies circulation in vampire bat populations using a betaherpesvirus-vectored transmissible vaccine. Proc. Natl. Ac. Sci. 2023; 120: e2216667120.

55. Almeida DS, Nogueira Paz L, Santos de Oliveira D, Nascimento Silva D, Ristow P, Hamond C, et al. Investigation of chronic infection by Leptospira spp. in asymptomatic sheep slaughtered in slaughterhouse. PLOS ONE. 2019; 14: e0217391.

56. Cordonin C, Turpin M, Bringart M, Bascands J-L, Flores O, Dellagi K, et al. Pathogenic Leptospira and their animal reservoirs: testing host specificity through experimental infection. Sci. Rep. 2020; 10: 7239.

57. Ratet G, Veyrier FJ, Fanton d'Andon M, Kammerscheit X, Nicola M-A, Picardeau M, et al. Live imaging of bioluminescent *Leptospira interrogans* in mice reveals renal colonization as a stealth escape from the blood defenses and antibiotics. PLOS Negl. Trop. Dis. 2014; 8: e3359.

58. Glennon EE, Becker DJ, Peel AJ, Garnier R, Suu-Ire RD, Gibson L, et al. What is stirring in the reservoir? Modelling mechanisms of henipavirus circulation in fruit bat hosts. Phil. Trans. R. Soc. B. 2019; 374: 20190021.

59. Gibson L, Puig Ribas M, Kemp J, Restif O, Suu-Ire RD, Wood JLN, et al. Persistence of multiple paramyxoviruses in a closed captive colony of fruit bats (*Eidolon helvum*). Viruses. 2021; 13: 1659.

60. Peel AJ, Baker KS, Crameri G, Barr JA, Hayman DTS, Wright E, et al. Henipavirus neutralising antibodies in an isolated island population of African fruit bats. PLOS ONE. 2012; 7: e30346.

61. Rahman S2, Hassan SS, Olival KJ, Mohamed M, Chang L-Y, Hassan L, et al. Characterization of Nipah virus from naturally infected *Pteropus vampyrus* Bats, Malaysia. Emerg. Infect. Dis. 2010; 16: 1990–1993.

62. Sohayati AR, Hassan L, Sharifah SH, Lazarus K, Zaini CM, Epstein JH, et al. Evidence for Nipah virus recrudescence and serological patterns of captive *Pteropus vampyrus*. Epidemiol. Infect. 2011; 139: 1570–1579.

63. Plowright RK, Foley P, Field HE, Dobson AP, Foley JE, Eby P, et al. Urban habituation, ecological connectivity and epidemic dampening: the emergence of Hendra virus from flying foxes (Pteropus spp.). Proc. Biol. Sci. 2011; 278: 3703–3712.

64. Becker DJ, Ketterson ED, Hall RJ. Reactivation of latent infections with migration shapes population-level disease dynamics. Proc. R. Soc. B. 2020; 287: 20201829.

65. Grinde B. Herpesviruses: latency and reactivation – viral strategies and host response. J. Oral. Microbiol. 2013; 5: 10.3402/jom.v5i0.22766.

66. Negrey JD, Thompson ME, Dunn CD, Otali E, Wrangham RW, Mitani JC, et al. Female reproduction and viral infection in a long-lived mammal. J. Anim. Ecol. 2022; 91: 1999–2009.

67. Butte NF, Wong WW, Treuth MS, Ellis KJ, O'Brian Smith E. Energy requirements during pregnancy based on total energy expenditure and energy deposition. Am. J. Clin. Nutr. 2004; 79: 1078–1087.

68. Speakman JR. The physiological costs of reproduction in small mammals. Philos. Trans. R. Soc. Lond. B Biol. Sci. 2008; 363: 375–398.

69. Plowright RK, Field HE, Smith C, Divljan A, Palmer C, Tabor G, et al. Reproduction and nutritional stress are risk factors for Hendra virus infection in little red flying foxes (*Pteropus scapulatus*). Proc. Biol. Sci. 2008; 275: 861–869.

70. Dietrich M, Kearney T, Seamark ECJ, Paweska JT, Markotter W. Synchronized shift of oral, faecal and urinary microbiotas in bats and natural infection dynamics during seasonal reproduction. R. Soc. Open Sci. 2018; 5: 180041.

71. Mourad M, Gershon M, Mehta SK, Crucian BE, Hubbard N, Zhang J, et al. Silent Reactivation of Varicella zoster virus in pregnancy: Implications for maintenance of immunity to varicella. Viruses. 2022; 14: 1438.

72. Drexler JF, Corman VM, Wegner T, Tateno AF, Zerbinati RM, Gloza-Rausch F et al. Amplification of Emerging Viruses in a Bat Colony. Emerg. Infect. Dis. 2011; 17: 449–456.

73. Encarnação JA, Dietz M, Kierdorf U. Reproductive condition and activity pattern of male Daubenton's bats (*Myotis daubentonii*) in the summer habitat. Mamm. Biol. 2004; 69: 163–172.

74. Martin L, Bernard RT. Endocrine regulation of reproduction in bats: the role of circulating gonadal hormones. In *Reproductive Biology of Bats*, (Elsevier) 2000; pp. 27–64.

75. Ruoss S, Becker NI, Otto MS, Czirják GA, Encarnação JA. Effect of sex and reproductive status on the immunity of the temperate bat *Myotis daubentonii*. Mamm. Biol. 2019; 94: 120–126.

76. Knowles SCL, Fenton A, Pedersen AB. Epidemiology and fitness effects of wood mouse herpesvirus in a natural host population. J. Gen. Virol. 2012; 93: 2447–2456.

77. Geiser F. Hibernation. Current Biology. 2013; 23: R188–R193.

78. Guerra-Silveira F, Abad-Franch F. Sex bias in infectious disease epidemiology: Patterns and processes. PLOS ONE. 2013; 8: e62390.

79. Zuk M. The sicker sex. PLOS Pathog. 2009; 5: e1000267.

80. Rossetto F, Iglesias-Caballero M, Liedtke HC, Gomez-Mestre I, Berciano JM, Pérez-Suárez G,et al. Mating strategy is determinant of adenovirus prevalence in European bats. PLOS ONE. 2020; 15: e0226203.

81. Sjodin AR, Willig MR, Anthony SJ. Quantitative Delineation of Herpesviruses in Bats for use in Ecological Studies. Ecology. 2019.

82. Ancillotto L, Russo D. Selective aggressiveness in European free-tailed bats (*Tadarida teniotis*): influence of familiarity, age and sex. Naturwissenschaften. 2014; 101: 221–228.

83. Ortega J, Guerrero JA, Maldonado JE. Aggression and tolerance by dominant males of Artibeus jamaicensis: strategies to maximize fitness in harem groups. J. Mammal. 2008; 89: 1372–1378.

84. Becker DJ, Bergner LM, Bentz AB, Orton RJ, Altizer S, Streicker DG. Genetic diversity, infection prevalence, and possible transmission routes of Bartonella spp. in vampire bats. PLOS Negl. Trop. Dis. 2018; 12: e0006786.

85. Foo YZ, Nakagawa S, Rhodes G, Simmons LW. The effects of sex hormones on immune function: a meta-analysis. Biol. Rev. 2017; 92: 551–571.

86. Klein SL. Hormonal and immunological mechanisms mediating sex differences in parasite infection. Parasite Immunol. 2004; 26: 247–264.

87. Baker KS, Suu-Ire R, Barr J, Hayman DTS, Broder CC, Horton DL, et al. Viral antibody dynamics in a chiropteran host. J. Anim. Ecol. 2014; 83: 415–428.

88. Breed AC, Breed MF, Meers J, Field HE. Evidence of endemic Hendra virus infection in flying-foxes (*Pteropus conspicillatus*)—Implications for disease risk management. PLOS ONE. 2011; 6: e28816.

89. Hayman DT, Luis AD, Restif O, Baker KS, Fooks AR, Leach C, et al. Maternal antibody and the maintenance of a lyssavirus in populations of seasonally breeding African bats. PLOS ONE. 2018; 13: e0198563.

90. Hasselquist D, Nilsson J-A. Maternal transfer of antibodies in vertebrates: trans-generational effects on offspring immunity. Phil. Trans. R. Soc. B. 2009; 364: 51–60.

91. Epstein JH, Baker ML, Zambrana-Torrelio C, Middleton D, Barr JA, Dubovi E, et al. Duration of maternal antibodies against Canine distemper virus and Hendra virus in Pteropid Bats. PLOS ONE. 2013; 8: e67584.

92. Alizon S. Co-infection and super-infection models in evolutionary epidemiology. Interface Focus. 2013; 3: 20130031.

93. Habig B, Chowdhury S, Monfort SL, Brown JL, Swedell L, Foersterh S. Predictors of helminth parasite infection in female chacma baboons (*Papio ursinus*). Int. J. Parasitol. Parasites Wildl. 2021; 14: 308–320.

94. Lunn TJ, Peel AJ, McCallum H, Eby P, Kessler MK, Plowright RK, et al. Spatial dynamics of pathogen transmission in communally roosting species: Impacts of changing habitats on bat-virus dynamics. J. Anim. Ecol. 2021; 90: 2609–2622.

95. Eby P, Peel AJ, Hoegh A, Madden W, Giles JR, Hudson PJ, et al. Pathogen spillover driven by rapid changes in bat ecology. Nature. 2022; 613: 340–344.

96. Markotter W, Vries LD, Paweska J. Wing tattoos: A cost-effective and long-lasting method for marking bats. Acta Chiropt. 2023; 25: 193–202.

97. Lebarbenchon C, Ramasindrazana B, Joffrin L, Bos S, Lagadec E, Le Minter G, et al. Astroviruses in bats, Madagascar. Emerg. microbes & infect. 2017; 6: 1–3.

98. Smythe LD, Smith IL, Smith GA, Dohnt MF, Symonds ML, Barnett LJ et al. A quantitative PCR (TaqMan) assay for pathogenic Leptospira spp. BMC Infect. Dis. 2002; 2: 13.

99. Tong S, Chern S-WW, Li Y, Pallansch MA, Anderson LJ. Sensitive and broadly reactive reverse transcription-PCR assays to detect novel paramyxoviruses. J. Clin. Microbiol. 2008; 46: 2652–2658.

100. VanDevanter DR, Warrener P, Bennett L, Schultz ER, Coulter S, Garber RL, et al. Detection and analysis of diverse herpesviral species by consensus primer PCR. J. Clin. Microbiol. 1996; 34: 1666–1671.

101. Wood SN, *Generalized additive models: an introduction with R* (CRC press). 2017.

102. Bonenfant C, Gaillard J-M, Dray S, Loison A, Royer M, Chessel D. Testing sexual segregation and aggregation: old ways are best. Ecology. 2007; 88: 3202–3208.

103. Streicker DG, Fallas González SL, Luconi G, Barrientos RG, Leon B. Phylodynamics reveals extinction–recolonization dynamics underpin apparently endemic vampire bat rabies in Costa Rica. Proc. R. Soc. B. 2019; 286: 20191527.

104. T. RStudio, RStudio: integrated development for R. *Rstudio Team, PBC, Boston, MA URL http://www. rstudio. com* (2020).

105. QGIS Development Team, QGIS Geographic Information System. Open Source Geospatial Foundation Project. (2022). Available at: http://qgis.osgeo.org [Accessed 27 July 2023].

**Figure 1. Widespread triple infection in *Mormopterus francoismoutoui.*** (A) Location of Reunion Island and (B) the studied roosts. (C) A picture of bats aggregated in the maternity roost (TBA site) before parturition. In (B) studied roosts are represented by grey circles on Reunion Island map and prevalence (white section of pie charts) is represented for paramyxovirus (in green), *Leptospira* bacteria (in red) and herpesvirus (in blue). A cross denotes the absence of prevalence data (no tested samples). Pie diameter corresponds to four categories of roost size: small < 500 individuals, medium: 500-2000 individuals, high: 2000-6000 individuals, and exceptional > 100,000 individuals. Accurate roost size estimation was not being performed for EGI and PSR roosts, but they are represented as medium roosts because several hundred/thousands are expected. Maternity roosts are underlined in pink. Roosts that were monitored only once are depicted by an asterisk. Maps were created using Qgis [105] with a background of OpenStreetMap under License CC- BY- S. This figure was inspired by Streicker et al. 2012 [48].

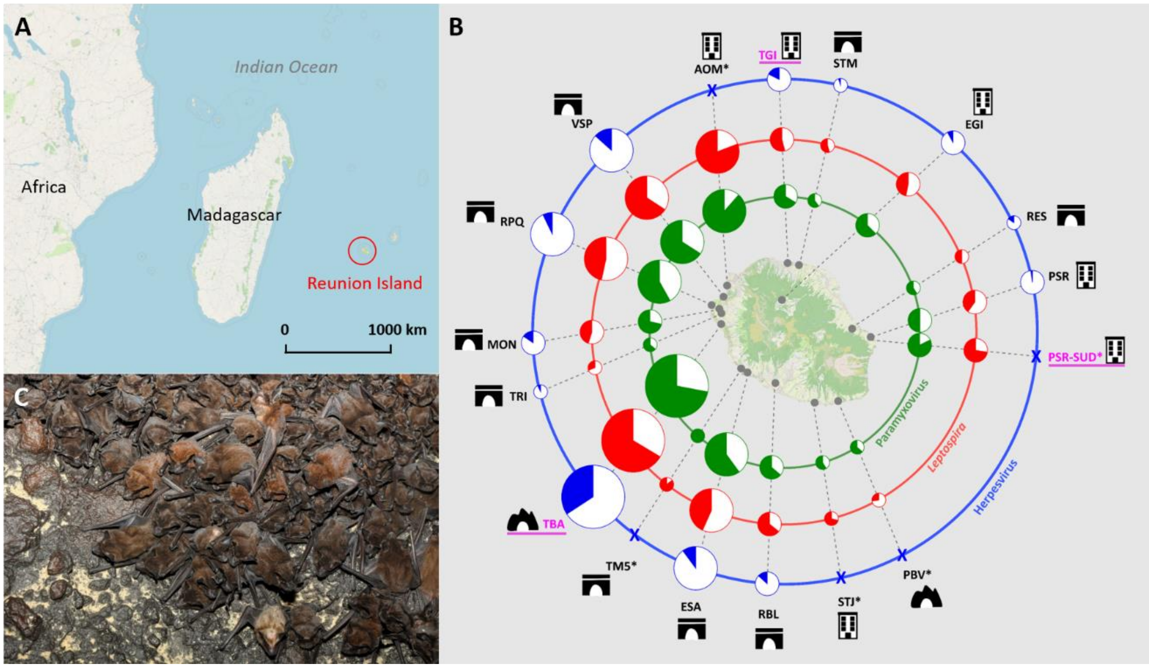

 Click here to access/download;Figure;Figure 2.pdf

**Figure 2. Age and reproduction are the main drivers of paramyxovirus, *Leptospira* bacteria and herpesvirus excretion dynamics in *Mormopterus francoismoutoui.***

(A) Observed prevalence in adults (solid line) and juveniles (dotted line), with grey areas representing 95% confidence intervals. Periods of significant change (where GAM derivative 95% does not overlap zero) are highlighted by light-colored segments (light green for PMV, orange for LEPTO and light blue for HPS). Arrows indicate periods where only one individual was tested. Pink and purple blocks at the bottom indicate the parturition period and the mating period, respectively. Austral summer and winter are indicated in yellow and blue, respectively. (B) GAMM-predicted prevalence in adults according to their reproductive status (active or not). Significant effects of GAMMs are indicated: *** $P < 0.001$ and ** $P < 0.01$.

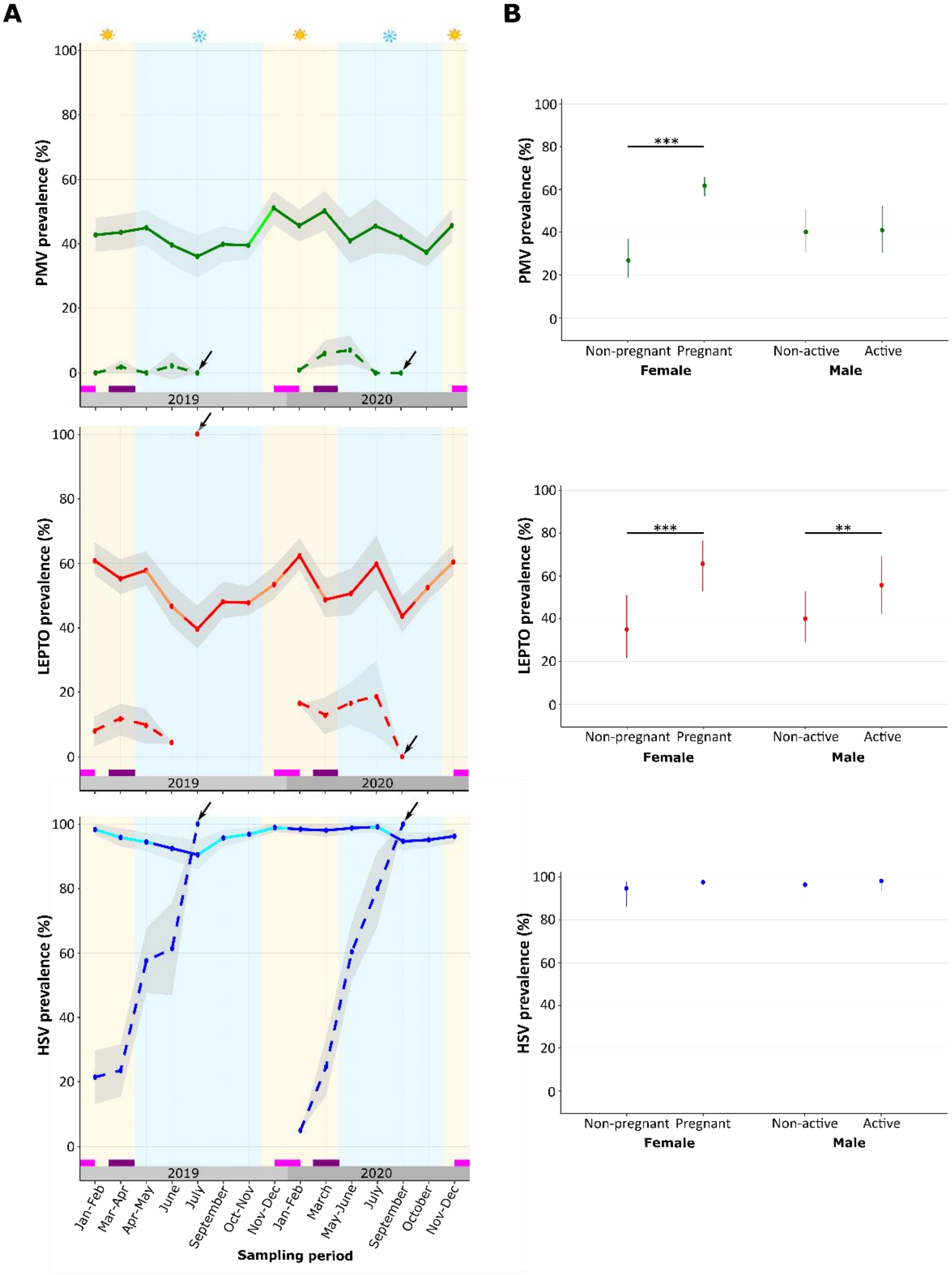

A
B
PMV prevalence (%)
LEPTO prevalence (%)
HSV prevalence (%)
2019
2020
Jan-Feb
Mar-Apr
Apr-May
June
July
September
Oct-Nov
Nov-Dec
Jan-Feb
March
May-June
July
September
October
Nov-Dec
Sampling period
***
**
Non-pregnant
Pregnant
Non-active
Active
Female
Male

Figure 3 Click here to access/download;Figure;Figure 3.pdf

**Figure 3. The probability of excretion increases with bi- and tri-infections.**

Infectious status is coded with 0 for non-shedding bats and 1 for bats shedding infectious agent(s). The 95% confidence interval is indicated by a black bar.

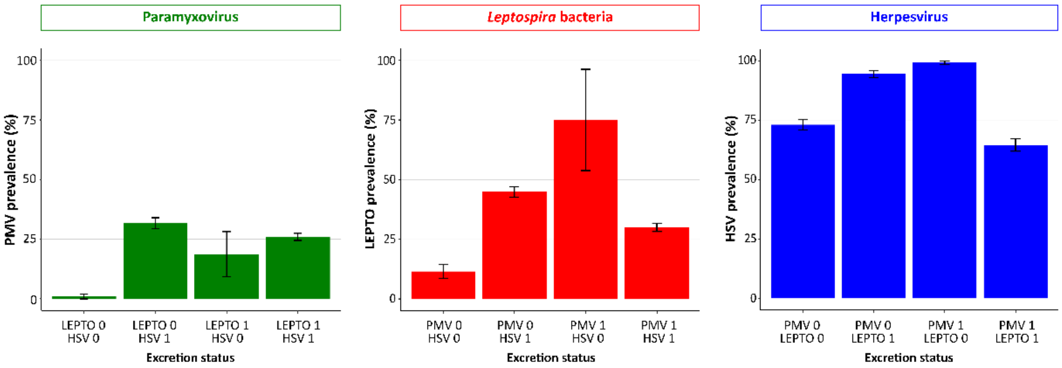

Figure 4

Click here to access/download;Figure;Figure 4.pdf

**Figure 4. Temporal changes of excretion status in recaptured *M. francoismoutoui* bats.** (A) Probability of remaining LEPTO and PMV positive through time. (B) Examples of recaptured bats with always-positive status for PMV and LEPTO. The time interval (in day) between two recaptures is indicated above the arrow. (C) Probability of the four categories of LEPTO excretion status change, with a 95% confidence interval in grey.

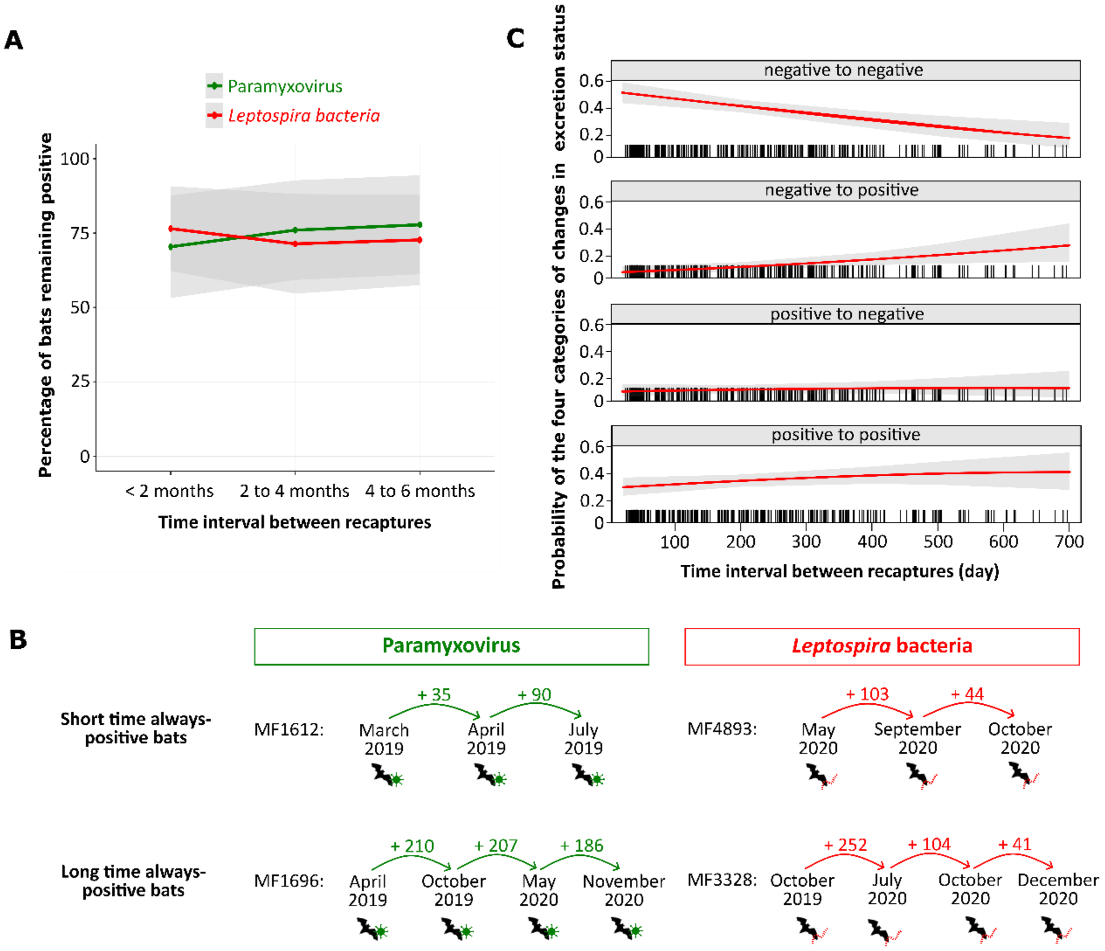

Click here to access/download
**Supporting Information**
AGUILLON_INFECTION_SUPPLEMENTALS.pdf
