# Supplementary material for: Role of individual and population heterogeneity in shaping dynamics of multi-pathogen shedding in an island endemic bat
Source: PLoS Pathog. 2025 Jul 11;21(7):e1013334. doi: 10.1371/journal.ppat.1013334 (PMC12273948; doi:10.1371/journal.ppat.1013334)
Supplement: Attachment — Submitted filename: PPATHOGENS-D-24-01674_letterresponse_R1.pdf [file ppat.1013334.s013.pdf]

Samantha Aguillon
UMR Processus Infectieux en Milieu Insulaire Tropical
2, rue Maxime Rivière 97490 Sainte Clotilde
Reunion Island
Email : samantha.aguillonfr@gmail.com

Sainte Clotilde, 12 February 2025

Dear Pr. Malim,

Please find with this letter, the revised version of our manuscript entitled "**Role of individual and population heterogeneity in shaping dynamics of multi-pathogen shedding in an island endemic bat**". We are grateful to the reviewers for their constructive comments on the initial submitted paper. We have carefully considered all remarks and modified the manuscript accordingly. You will find the reviewer's comments and our replies at the end of this letter.

All authors have participated in revising the manuscript and have agreed on its resubmission to *Plos Pathogens.*

Thank you for your consideration of our work.

Sincerely yours,
Samantha Aguillon, on behalf of the other authors

Reviews of Manuscript : PPATHOGENS-D-24-01674R1

Please note that author's responses follow each reviewer's comment and start with ">>>". Line numbers correspond to the clean version of the manuscript.

Part I – Summary

Reviewer #1: Aguillon et al present an extensive longitudinal study on the simultaneous excretion of paramyxoviruses, herpesviruses and Leptospira bacteria in the Reunion free-tailed bat across 17 roosts over 24 months. The authors detect year-round excretion at high prevalence, with little temporal synchrony among roosts. Longitudinal studies of infectious agents in bats are still rare and so this study presents a valuable contribution. In particular, the multi-agent focus here is particularly rare, as is the high recapture rate of individuals. The latter is almost unheard of in bat studies. This allowed the detection of individuals that are infected across multiple sampling periods - interpreted here as persistent infections and cycles of clearance and reinfection. If this is indeed the case, it provides critical data on mechanisms of viral maintenance in bat populations - a major point of conjecture in this field.

# R1.1: One critical gap in the analyses is analyses and a description of whether the detections here represent multiple species, variants or strains of each of the viral families or the bacteria genus. The analyses implicitly imply that it is a single paramyxovirus species, a single herpesvirus species, and a single species of Leptospira, however I did not see this examined or even if the PCR was family-level or species specific. This should be clarified explicitly and given that the entire interpretations rest on this assessment, this would be imperative to be addressed by revisions. Also, even the detections do represent single species within each family, it is a shame not to see any genetic analyses to confirm whether these repeated positive detections are persistent infections or re-infections. While persistent infections of herpesviruses (and I think Leptospira) are well known, evidence for the same for paramyxoviruses is more circumstantial. Inclusion of such data would be a huge boost to this study and the inferences that can be made. I would strongly encourage the authors to consider adding that work to this manuscript - with it, I think this paper could emerge as a foundational study for future work in the field.
>>> In response to the reviewer's comment, we have added more details to the methods section, specifying that PCRs used for detection were broad-spectrum for each pathogen, allowing the identification of multiple species/variants (page 22: lines 553-555 and lines 558-559).

We agree with the reviewer that detecting different species/variants is crucial for understanding infection dynamics. This has recently been demonstrated for coronaviruses (Geldenhuys et al., 2023) and paramyxoviruses (Mortlock et al. 2021, Peel et al. 2019). In Reunion free-tailed bats, we have previously shown the presence of several paramyxovirus variants, but only one variant of *Leptospira* circulating within a single colony (Dietrich et al. *2015*). For this reason, we performed Sanger sequencing on some PCR-positive paramyxovirus samples of the present study, representing only a small portion (n=200) of the high number of positive samples (n=2000), due to budget constraints (the project and its funding have ended). The sequences confirmed the presence of two distinct PMV variants. These data are currently being combined with those from a four-year longitudinal study (Hoarau et al. 2022) and will be published soon, explaining they are not presented in the present study. Meanwhile, we are seeking additional funding to complete the sequencing of the remaining positive samples.

For *Leptospira*, obtaining sequencing data from urine samples is challenging due to the low DNA yield. For example, in a previous study, we reported a 20% failure rate (Dietrich et al. 2018). Moreover, sequencing multiple genes (via the MLST scheme) is required to differentiate between species/variants, further increasing the cost. As a result, we have not yet sequenced the *Leptospira*-positive samples from this study.

Regarding herpesviruses, we suspected the presence of several variants, as co-infections were recently identified in vampire bats (Griffiths et al. 2020) and some of our preliminary data with Sanger sequencing showed double peaks on the chromatograms. Therefore, we employed an Illumina sequencing approach on a smaller number of bats with recaptures to track individual shedding histories. The sequences (n=121) revealed 20 variants across the alpha, beta, and gamma herpesvirus subfamilies. A manuscript on these findings is now available as a preprint (Aguillon et al. 2025) and will be submitted in a peer-reviewed journal. Given the partial nature of the sequencing data produced so far, analyzing shedding dynamics by variant is not yet feasible but will be possible once additional funding is secured. However, we now discussed our preliminary genetic data (page 16: lines 398-403), emphasizing the challenges posed by the occurrence of multiple variants in interpreting global prevalence and highlighting the need for further sequencing efforts (pages 16-17: lines 403-408).

Aguillon S, Turpin M, Le Minter G, Lebarbenchon C, Hoarau AOG, Mavingui P, Dietrich M. Bat things come in threes: within-host dynamics of herpesvirus triple infection in bats. bioRxiv 2025.02.05.636680.

Dietrich M, et al. 2015. *Leptospira* and Paramyxovirus infection dynamics in a bat maternity enlightens pathogen maintenance in wildlife. *Environmental Microbiology* 17: 4280–4289.

Dietrich M, et al. 2018. Biogeography of Leptospira in wild animal communities inhabiting the insular ecosystem of the western Indian Ocean islands and neighboring Africa. *Emerging Microbes & Infections* 7:57.

Geldenhuys M, et al. 2023. Viral maintenance and excretion dynamics of coronaviruses within an Egyptian rousette fruit bat maternal colony: considerations for spillover. *Scientific Reports* 13: 1–13.

Griffiths ME, et al. 2020. Epidemiology and biology of a herpesvirus in rabies endemic vampire bat populations. *Nature Communications* 11: 1–12.

Hoarau AOG, et al. 2023. Synchronicity of viral shedding in molossid bat maternity colonies. *Epidemiology and Infection* 151: e47.

Mortlock M, et al. 2021. Seasonal shedding patterns of diverse henipavirus-related paramyxoviruses in Egyptian rousette bats. *Scientific Reports* 11: 1–12.

Peel AJ, et al. 2019. Synchronous shedding of multiple bat paramyxoviruses coincides with peak periods of Hendra virus spillover. Emerg. microbes & infect 8: 1314–1323

# R1.2: Identifying the drivers of patterns in excretion remains challenging - here, it seemed difficult to ascertain age in some months, and the proportion of juveniles captured was very low (with misclassification as an identified issue). This is concerning given the results suggest that transmission is highly dynamic in this cohort - at least for HSV. Rigorous assessment of the statistical approaches is outside of my expertise, however I suspect that there could be some issues there - e.g with the low % of explained deviance in some of the GAMMS, and the absence of model comparisons e.g. through AIC. Other reviewers may be able to provide more specific feedback here. >>> The discussion about misclassification of juveniles has been strengthened in different parts of the manuscript (page 11: lines 240-243, page 11: lines 252-258, pages 18-19: lines 465-470, page 24: lines 619-630). Statistical analyses have been extensively reviewed and completed, according to detailed suggestions of reviewer 2. The AIC was not used for GAM selection due to the complexity of the model (non linear terms and interactions) which make AIC less reliable for evaluating their performance. Instead, we used the gam.check function to evaluate model validity, including checks on the residual distributions, the adequacy of the smoothing basis (k diagnostics), and overall model fit. Conversely, we used the AIC criterion for GLM selection, as these models were less complex.

# R1.3: The rates of co-excretion of pathogens is also exceptional. In the co-infection results section, I would have liked to see more attention to the nature of the bi-infecting agents e.g. bacteria-virus co-infections versus virus-virus co-infections, and whether the nature of the bi-infection affected the likelihood of tri-infection. I had trouble interpreting the figure. I think this is an incredible dataset that may warrant more sophisticated co-occurrence analyses that takes dependencies into account.
>>> Thank you for recognizing the potential of our dataset. We have updated the analyses to go deeper into the analysis of coinfections (page 13-14: lines 272-277). Specifically, we added an interaction term between infectious agents in the GAM models M1, M2, and M3 to assess how bi-infections influence the likelihood of tri-infections. Additionally, we have revised Figure 4 to enhance clarity.

# R1.4: Overall, I'd like to encourage the authors - The major contributions of this study are the longitudinal dataset, high rates of recapture and repeated positivity, and the high rates of co-infection. These factors provide the opportunity for major insights into the mechanisms driving pathogen transmission in bat populations. However, I feel that the current analyses and clarity of writing are not rigorously assessing and clearly communicating the value of this dataset and these results. I would like to see a revision that addresses these points.
>>> We appreciate the reviewer's positive feedback and words of encouragement. We have made significant revisions to both the analyses and the manuscript text to ensure a more rigorous assessment and clearer communication of our findings. We hope these updates now better emphasize the unique contributions of our dataset.

Reviewer #2:
#R2.1: This manuscript examines patterns of pathogen shedding for three pathogens (Herpes and Paramyxovirus viruses and Leptospirosis bacteria) over space and time (12 roosts sampled ~15 times, and 5 sampled once) in of a single species of bats on an oceanic island. The dataset is moderately large (3700 samples overall, but only ~20/site-sampling period). This type of dataset is extremely rare and contrasts markedly with anecdotal sampling often carried out when sampling bats. Further, the study examines pathogen shedding (rather than serology) which is especially interesting since it is of more importance for transmission.
The broad patterns are very interesting – shedding of HSV is nearly complete (87% of all bats), whereas shedding of the other two pathogens is very high (30-50% of all bats). This contrasts strongly with some other bat pathogens in which shedding is rarely detected (e.g. Henipaviruses, Filoviruses, Lyssaviruses). Temporal patterns of shedding are only weakly interesting, likely due to the very short study period (24 months). The spatial patterns aren't examined in any meaningful way except a poorly executed synchrony analysis (see below for details). Coinfection patterns are likely quite interesting, but the analyses and data presentation don't allow the reader to assess the data properly. There were some differences in shedding with individual traits (e.g. sex, age, reproductive status), but some of these analyses were problematic and likely spurious (details below).
Overall, the study has a very nice data set, but the analyses were highly problematic (extensive details below) which makes the conclusions unsupported. If the authors revise the analyses extensively, this could be a very important contribution to our understanding of pathogen dynamics in bats.
>>> Thank you for your detailed feedback and constructive critique. We have extensively revised our analyses to address the concerns raised and to ensure that our conclusions are robust and well-supported. Below, we provide detailed responses to each of your points.

Part II – Major Issues: Key Experiments Required for Acceptance

Reviewer #1:
# R1.5:- sequencing to confirm viral/bacterial species and that there are in fact not multiple co-circulating paramyxoviruses, multiple co-circulating herpesviruses and multiple co-circulating Leptospira species
- to fully justify claims of persistent infections, sequencing of sufficient depth to demonstrate identical strains within the same individual over time, subject to minor within-host evolution
>>> Please see our previous response (comment #R.1.1).

Reviewer #2: Major statistical issues:
#R2.2: - The paper doesn't actually show any of the statistical models including coefficients, SEs, P-values, etc. Add a supplemental table with full details for each of the models M1-M3, and M10-M13, and ideally for all the other models as well. The table should indicate the details of the statistical model (what distribution was used for the response? What link function?), the full results (for each predictor include the coeff, SE, Z/t value and exact P-value (NOT <0.05)). Indicate the variance/deviance explained of the model and compare this to a null model that includes the same random effects so it's clear how much additional variance/deviance the fixed effects are adding.
>>> We appreciate the reviewer's insightful comment. In response, we have provided comprehensive supplemental tables (Tables S1, S2, S3, S4 and S5) that summarize the full results for each model and include all the requested details for the predictors. Additionally, aligning with the reviewer's suggestions regarding the factors to include in the models (see #R2.3 and #R2.34), we have updated the models, and they no longer include random effects. This modification simplifies the statistical framework and ensures that all predictors are evaluated consistently across the dataset, but we noticed that the deviance explained is roughly similar to our first models. Since there is no more random effects, we did not perform the comparison with the null model for GAM as the deviance explained by fixed effect is calculated in the GAM function. However, for GLM we did compare full model with null model. In both case, we reported the percentage of variance explained in the tables in supp.

#R2.3: - The analyses are set up in a way that makes it unlikely they will detect some effects even if they are present and strong (e.g. colony size – see below).
- They analyses include random effects for things that shouldn't be random (e.g. there are only 2 years of data and yet "year" is a random effect; one can't/shouldn't estimate a variance with 2 data points).
- The fitted models have many redundant terms – e.g. sampling period and julian date (the latter as a random effect which means it will be treated as a factor, not a continuous variable). These are both explaining the same thing (temporal patterns) and the inclusion of both obscures the actual explanatory power of each one.
>>> Thank you for pointing out these important concerns regarding the model structure. We have revised the models accordingly, by removing 'Year' as a random effect and by retaining "sampling period" as a smooth function to address temporal patterns more effectively and avoid redundancy with other variables (see Tables S1 and S3).

#R2.4: - The paper argues there was no synchrony in pathogen dynamics among roosts, when the study length is simply far too short (2 yrs) to detect it. Further there was actually evidence of synchrony for one of the 3 pathogens despite the very short study duration suggesting synchrony is likely much higher and would be easily detectable across reasonable time periods. Further the

analysis the authors use, a rank test, is a terrible way to analyze this data because it has very little power.
>>> We acknowledge that the dataset covers only two years, but we conducted multiple sampling sessions throughout each year: 8 times in the first year and 7 times in the second, leading to 15 distinct sampling points. Consequently, we assessed pairwise synchrony of prevalence across these 15 sampling points.
As suggested by the reviewer, we replaced Spearman's rank correlation with Pearson's correlation to avoid potential issues associated with rank-based tests (page 22: lines 575-577). While the reviewer did not specify a particular type of analysis, we performed a new analysis using spline correlograms to visualize the relationship between synchrony and geographical distance. We also explored Dynamic Factor Analysis (DFA), which is well-suited for identifying common trends in short, nonstationary time series (page 23: lines 584-589). These new analyses provide more evidence of synchrony, with slightly stronger synchrony observed for HSV compared to PMV and LEPTO. However, a longer time series could potentially enhance the detectable signal. Consequently, we have modified our results (page 9: lines 195-211) and discussion (pages 14-15: lines 347-352).

#R2.5: - Overall, it's quite disappointing that the authors don't take more advantage of the data and fit mechanistic models (such as those in Brook et al 2019 J. Animal Ecology, Epstein et al 2020 PNAS) so they can learn much more about the biology underlying the data. The GAMMS used here are a very poor substitute.
>>> We agree that mechanistic models would be highly valuable and are indeed a powerful tool for understanding the underlying biology. However, developing and applying such models is beyond the scope of the present study, which focuses on descriptive analyses of shedding dynamics, and GAMMs are a well-established initial approach, as also employed in the above cited papers by Brook et al. (2019) and Epstein et al. (2020).
At this stage, our dataset has limitations that preclude the effective parameterization of robust mechanistic models, particularly in terms of detailed demographic data across multiple colonies. We have begun to address these limitations by collecting long-term capture-mark-recapture (CMR) data to improve demographic estimates. Additionally, a separate study focusing on mechanistic modeling within a single roost is currently in development (forthcoming work in Hoarau's PhD thesis).
Once these demographic data are sufficiently mature, they will be integrated into spatially-explicit mechanistic models that encompass multiple colonies. The discussion now emphasizes the necessity of such models in future studies to unravel the mechanisms underlying our findings (page 18: lines 437-443).

Hoarau AOG, 2022. Viral co-infection dynamics in bats in the Western Indian Ocean. PhD Thesis. University of Reunion Island.

#R2.6: Data presentation :The data presentation is generally poor. None of the figures that show the shedding prevalence data (e.g. Fig 2A, Fig S2) show the uncertainty in the prevalence. The figures show the model uncertainty but that is quite different from the binomial uncertainty for a given sample. Sample sizes are never shown on any of the figures. Add them to the figures.
>>> Figures have been updated to include both the raw data and model outputs as requested, and the uncertainty in prevalence. Additionally, sample sizes have been added to the figures in general.

#R2.7: Language issues :The paper needs to be read by a native speaker. It contained dozens/hundreds of grammatical issues, misuses of words, and was quite difficult to understand.
>>> The paper has been carefully reviewed and corrected.

#R2.8: Logical/conceptual issues : The response variable the study measured – pathogen shedding – is not the same thing as infection, but in many places the authors appeared to treat the two as the same. They are not the same and comparisons between studies that measured either infection or measures of past infection (e.g. serology) cannot be easily compared. One wouldn't expect the same patterns over time or among individuals or ages.
>>> We have revised the text and now use the term "shedding" for our results.

#R2.9: Finally, there is a huge unanswered question that the authors strangely don't address. They show patterns of pathogen shedding for adults and juveniles. For 2 of the 3 pathogens shedding prevalence in juveniles is a tiny fraction of that in adults. It's not clear when juveniles get infected and start shedding at prevalences that match the adults. The gap between juveniles and adults likely explains several of the patterns the authors attribute to other factors (e.g. the effect of reproduction – see below). Most worrisome, it appears the authors weren't able to age bats during the key part of infection and shedding (after 6 months of age), making the overall patterns potentially simply a result of including a variable number of old juveniles in different samples. Can the authors use the size measurements (e.g. forearm length, mass) plus recaptures to identify the ages of individuals more carefully?
>>> Unfortunately, there is actually no validated morphological measure available to distinguish between old juveniles and adults in the field. After approximately six months of life, the size of the forearm length of juveniles is identical to that of adults (Aguillon et al. 2023: Figure S6).

If misclassified juveniles were responsible for the lower PMV and LEPTO prevalence observed in non-reproductive bats, it would imply that « old » juveniles [~11 months old in November (gestation), 15 months old in March (mating)], are still uninfected. This would suggest that maternal antibodies last for a long period in this species. This hypothesis is now clearly mentionned in the manuscript (page 15: lines 365-373). To provide more information, we have re-analyzed data by comparing shedding in females with different nipple development stages. In particular, we looked at non-pregnant females classified as adults (based on fused epiphyses) but lacking developed nipples (M0), which may include both sexually mature adult females that have not yet reproduced, and sexually immature (misclassified) juveniles. Those females exhibited a lower shedding prevalence, compared to those with developed nipples. For males, we don't have any other morphological data that could discriminate old juveniles and adults during the mating period. We have highlighted this limitation in the methods (page 24: lines 619-630), results (page 11: lines 252-260) and in the discussion (page 15: lines 368-373) and mentioned the importance of serological data in estimating the duration of maternal immunity in this bat species (page 15: lines 373-374, page 16: lines 386-388).

Aguillon S, et al. 2023. A population in perpetual motion: Highly dynamic roosting behavior of a tropical island endemic bat. Ecol. Evol. 13: e9814.

Part III – Minor Issues: Editorial and Data Presentation Modifications

Reviewer #1:
# R1.6: In the attached marked up PDF, I have highlighted some sentences where the meaning was not clear, and made some suggested minor changes to wording throughout. I have also underlined sections in where the phrasing wasn't clear and could be improved or the grammar was incorrect. General editing for clarity and grammar would be helpful, however overall, the ideas and intended meaning was mostly clear and the structure was logical. Apologies if some of my comments are addressed within the manuscript - hopefully revision of the text will help make these points more explicit and clear.

>>> We have addressed the requested modifications within the manuscript.

# R1.7: Some additional points are below (in addition to those in the PDF):
- The disparity between the number of juveniles caught and adults caught suggest to me that there is misclassification occurring. This is mentioned at one point, however I think that it it needs to be more clearly highlighted as a limitation to interpretation in the results and the discussion
>>> We have emphasized this point in the methods (page 24: lines 619-630), results (page 11: lines 254-258) and in the discussion (page 15: lines 368-373)

# R1.8: - Further discussion of the consistency of the minimal detections in juveniles with previous studies in other bat species is warranted
>>> We have expanded this section in the revised manuscript to emphasize these findings and how they compare with the broader literature (page 15: lines 368-373).

# R1.9: - linking density with roost size is problematic. An initial assumption in then comparing this with prevalence (and making statements about density dependence) is that roost size correlates with density. Is there evidence that that is the case? ie. that the density of individuals per sq-m changes as the population grows? If these data are not available, then references to density should be removed, and just focus on roost size. Density and roost size are frequently conflated in viral transmission studies in bats.
>>> Estimating the density of bats within their roosts presents significant challenges, as it often involves photos or videos of bats that may be roosting in extremely confined spaces. Consequently, we currently lack data demonstrating a clear positive correlation between roost size and the density of bats. In light of this limitation, we have opted to use the term *roost size* rather than *density* throughout the text to better reflect the available data and avoid potential misinterpretations.

# R1.10: - With the high prevalence rates and inability in telling apart persistent infection versus re-infection, I would have thought that it would be challenging to confidently assess synchrony and metapopulation dynamics. I think this needs to be toned down or further justified.
>>> We have thoroughly reviewed the synchrony analysis; please refer to our response above for comment #2.4.

# R1.11: - I found the discussion around reactivation of PMV and Leptospira infections during pregnancy interesting, but unconvincing given the lack of seasonality in infections. I would like to see versions of figure 2B that incorporate time - i.e. can you compare pregnant versus non-pregnant females during the season when most female bats are mid pregnancy. Also, given the challenge in differentiating first year juvenile bats of adult size at this time, how well can this effect be separated from age? Again, sequencing of positive recaptured bats would be a huge contribution to these inferences.
- overall I felt that the discussion was too speculative. I would suggest focusing only on the more strongly supported results, aiming for an integration of the discussion points in a way that pulls together the most parsimonious hypotheses for the whole study. Currently, there are many disconnected mechanisms discussed to explain individual results, without a strong connected thread throughout the discussion
>>> Thank you for your suggestion to incorporate time into Figure 2B. We have conducted the analysis to compare pregnant versus non-pregnant females over time (see figure below), but it did not provide additional insights that would substantively enhance the manuscript. Furthermore, when analyzing data by date, the number of non-pregnant females was too low to provide robust

estimates, resulting in very large confidence intervals. For these reasons, we have opted not to include these results in the final version of the paper.
>>> We have added a paragraph in the discussion for the potential bias induced by misclassification of juveniles as adults (see also our response to comments #R2.9).
>>> As suggested, some parts of the discussion have been removed or shortened to focus on the strongly supported results.

PMV : 2019 and 2020

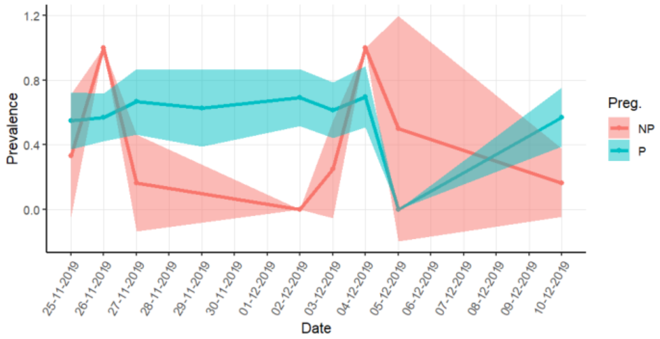

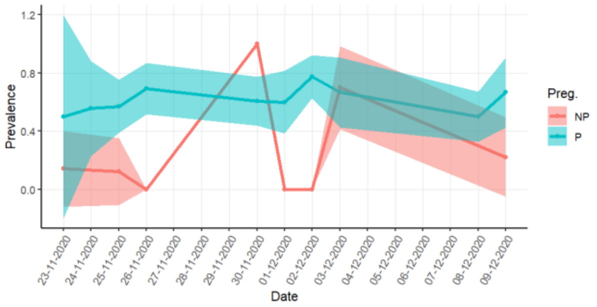

LEPTO : 2019 and 2020

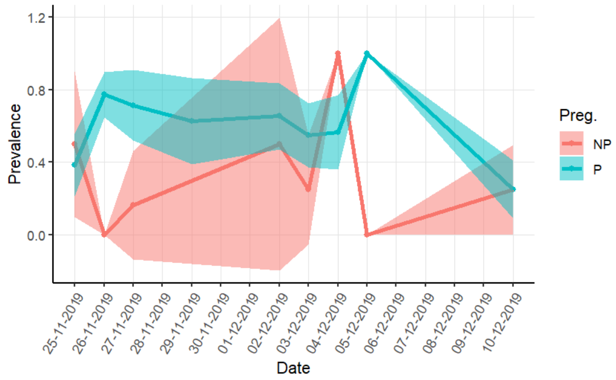

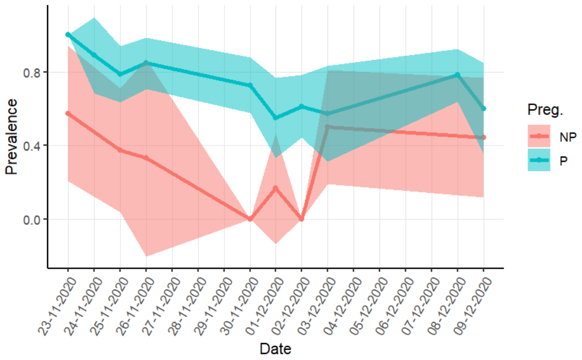

HSV : 2019 and 2020

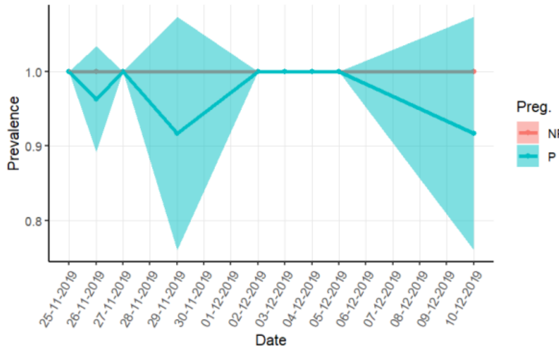

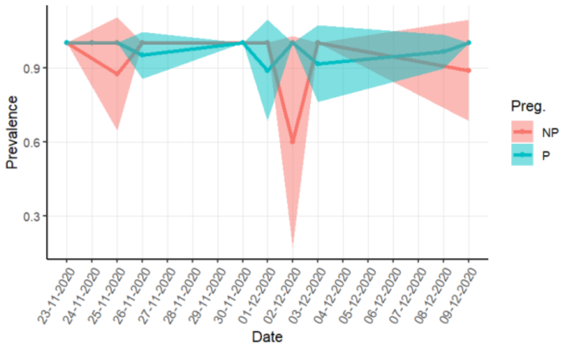

<u>Reviewer #2:</u> These are NOT minor comments. They are detailed comments pertaining to individual parts of the paper.
Detailed comments

#R2.10: L58-60 This sentence contradicts itself. Infection is either persistent or gets cleared. It can't be both.
>>> The abstract has been reviewed and highlights that mechanisms of persistence remain to be identified (page 3: lines 61-62).

#R2.11: L62 Do you mean "recrudesce"?
>>> The sentence has been changed. See our comments above.

#R2.12: L49-70 There are many other studies like this one but the authors make no effort in the abstract to acknowledge this work, build on it and indicate what new has been learned from this study. I'd suggest condensing the current abstract to 2/3 of it's current length and adding both an introductory sentence that lays out what currently isn't known and then 1-2 concluding sentences that indicate how the results from this study further our general knowledge. Otherwise. the work is only of limited interest outside of Reunion.
>>> We have changed the abstract to more clearly emphasize the originality of our study in comparison to previous research (page 3: lines 51-53).

#R2.13: L75 How is it a "model"?
>>> As emphasized in the introduction, small island populations—particularly island endemic species—offer ideal natural settings for studying infection dynamics at the population level. Their closed geographic boundaries and reduced interspecific interactions (lower species richness) make them especially suitable for such studies. To enhance clarity, we have updated this section of the introduction (page 6: lines 113-122) and revised the author summary to avoid potential confusion (page 4: line 76).

#R2.14: L79-80 See comment above. This is a self contradiction.
>>> As previously shown, both persistence and clearance mechanisms can occur within the same bat population (e.g. Glennon et al. 2019), so we believe that presenting both is not necessary contradictory. Our data suggest this is the case for PMV in Reunion free-tailed bats, as noted in the discussion (page 17: lines 410-418). However, since we could not confirm the specific mechanisms driving the observed shedding dynamics, we have removed this point from the author summary and emphasized the need for further studies to identify these mechanisms in the discussion (pages 18: lines 437-443) and abstract (page 3: lines 61-62).

Glennon EE, et al. 2019. What is stirring in the reservoir? Modelling mechanisms of henipavirus circulation in fruit bat hosts. Phil. Trans. R. Soc. B. 374: 20190021.

#R2.15: L82 Really? Most other studies suggest reducing spillover primarily involves reducing human-wildlife contact.
>>> We agree that minimizing human-wildlife contact is a cornerstone of spillover prevention, however interventions aiming at reducing human-wildlife contact could be strategically enhanced by targeting high-risk periods, that correspond to shedding pulses (Becker et al. 2019). The author summary has been re-written (page 4: lines 82-84).

Becker DJ, et al. Ecological conditions predict the intensity of Hendra virus excretion over space and time from bat reservoir hosts. Ecol. Lett. 2023; 26: 23–36.

#R2.16: L85 There is no definitive evidence that SARS-CoV-2 is a bat virus. Revise this sentence to reflect this. There are many other pathogens where the reservoir hosts are known (e.g. SARS-CoV-1, Nipah virus, rabies virus, etc.). Use one of these instead.

>>> We did not intend to suggest that SARS-CoV-2 is a bat virus, but rather to emphasize its relationship to a bat virus (closest relatives to date are in bats, Temmam et al. 2022), which highlights the importance of understanding virus transmission in bats. We acknowledge that the original sentence may have been unclear and have revised it accordingly (page 5: lines 85–88).

Temmam S, et al. 2022. Bat coronaviruses related to SARS-CoV-2 and infectious for human cells. Nature 604: 330–336.

#R2.17: L88 Ref 4 is a review and has no original data, so it's not a great ref for this statement. There is no statistical analysis linking spillover to shedding in ref 2. Finally, the analyses in Ref 3 linking shedding to spillover are quite problematic – the data are pseudoreplicates and are analyzed a half dozen ways without any attempt to control for multiple comparisons. If there are other papers that show links between shedding and spillover, please cite them here instead of refs 2-4.
>>> We modified the references accordingly (page 5: line 89).

#R2.18: L92 There are several robust datasets that must be acknowledged here, including ref 11, as well as work by Heymann et al., Brooks et al JAE, etc.
>>> We have chosen to cite Becker et al. 2019 because they conducted a comprehensive meta-analysis of spatiotemporal studies. However, we now include supplementary papers a requested, to provide examples of recent robust datasets (page 5: line 93).

#R2.19: L106 Add a key reference on coinfections: Graham 2008 PNAS www.pnas.org cgi doi 10.1073 pnas.0707221105
>>> Thank you for this suggestion. We added it (page 5: line 107).

#R2.20: L115 Replace "flight" with "fly"
>>> Corrected (page 6, line 116).

#R2.21: L117 do you mean "on islands"?
>>> This has been changed by "60% inhabit islands" (page 6, lines 117-118).

#R2.22: L188 Replace "help sample" with "enable sampling"
>>> Changed (page 6: line 119).

#R2.23: L112-132 This paragraph adds nothing to the paper and should be deleted
>>> Conducting longitudinal studies can be challenging due to the difficulty of obtaining sufficient samples. However, we emphasize that the Reunion free-tailed bat is a particularly interesting species for such studies because it is both abundant (making colonies easy to locate) and hosts large populations (facilitating the collection of reliable sample sizes from each colony at multiple time points). Therefore, we chose to retain this paragraph and have made several revisions to enhance its clarity (page 6: lines 116-135).

#R2.24: Figure 1 – why are you showing prevalence with the white portion of pie charts? This is counterintuitive and will confuse the reader. Please use the color for prevalence. Even better, use a bar chart rather than a pie chart. Pie charts are well known to be misleading for visualizing data. See, for example:
https://scc.ms.unimelb.edu.au/resources/data-visualisation-and-exploration/no_pie-charts
>>> We revised the figure by applying the reverse color scheme. However, we retained pie charts instead of switching to bar charts, to keep the relationship between pie diameter and roost size.

#R2.25: Fig 2. Are you really showing GAMMs? It looks like you are just connecting the prevalence with lines and not showing binomial sampling error for each point (nor sample size).
>>> The graph has been updated to display both raw data (with sample size) and model outputs (with binomial sampling error), now corresponding to Figure 3.

#R2.26: Fig 2. Please add letters to all panels of the graph, not groups of panels.
>>> Done (Figure 3).

#R2.27: Fig 2. For the HSV prevalence panel (bottom right) there is so much whitespace that it's not possible to see the differences (or lack thereof) among the groups. Can you change the y-axis limits to be 80 – 100?
>>> In order to facilitate comparison with the prevalence of PMV and LEPTO, and to highlight the very high prevalence of HSV, we prefer to keep the same scale. Additionally, with the inclusion of results for females M0 and their wide confidence intervals, reducing the y-axis is no longer possible. The differences are indicated with stars between the groups (Figure 3).

#R2.28: Fig S1 legend Replace "is resumed" to "is shown"
>> This figure has been revised and is now Figure 2. We have also modified the legend and the term 'is resumed' is no longer used.

#R2.29: Fig S1 If the goal is to examine synchrony in infection dynamics of individual pathogens among roosts then it would be better to make 3 graphs – one for each pathogen – and show all the roosts on that graph. It's currently very difficult to see the synchrony or lack thereof in Fig S1.
>> We have revised the graphs and now present a separate graph for each pathogen (see Figure 2).

#R2.30: L187 What is M1? You need to tell the reader what the M1, M2, etc. are since the results appear before the methods.
>>> M1 refers to the model's number detailed in Table S1-S5. We have modified the sentences accordingly (see for example page 10: lines 215-216).

#R2.31: L202 Overall synchrony may be low, but it appears to be higher for Herpesvirus, with 4 of the roosts being synchronous, whereas only one pair of roosts was synchronous for paramyxo.
>> We agree with the reviewer that there is a slight difference in synchrony, which we now emphasize in the text along with the results of the new analyses performed (pages 9-10: lines 195-211), especially the regional synchrony calculated with the *sncf* package which indeed supports a higher level of synchrony for herpesvirus.

#R2.32: L208 Report the P-values. Don't just say P<0.05. There is nothing magical about 0.05.
>>> We have modified the presentation of the p-values throughout the entire manuscript.

#R2.33: L208 and Results: Where are the details of the statistical analyses? You MUST provide a table of the fitted models with coefficients, SEs, etc. A set of supplemental tables would be fine.
>>> Details of the models are now included in supplementary information (Tables S1 to S5).

#R2.34: L208 If you are using models M1-M3 to make this inference, it is highly problematic. You've included Site as a random effect in these models which means that any variation among sites in roost size would be mostly captured by the random effect and not by roost size. As a result, you're

basically just analyzing variation in roost size within sites over time and variation in prevalence over time is already captured by your Sampling Period predictor. In short, you didn't find a roost effect because you included other variables that make it redundant and thus non-significant. Here's a super simple simulation in R that shows this:

```
set.seed(5)
df=data.frame(mRS=rep(10^rnorm(17,3,1.),each=15),site=rep(LETTERS[1:17],each=15),
month=rep(1:15,17) ) #17 sites, 15 points each, with mean roost size varying
df$tRS=rnorm(nrow(df),df$mRS,.2*df$mRS) # add temporal variation around mean roost size
ggplot(df,aes(x=month,y=tRS,color=site))+theme_few()+ #plot roost size vs time for sites
geom_point()+geom_line()+
scale_y_continuous(trans="log10")
df$prev=rnorm(nrow(df),.3+.02*log10(df$tRS),.1) #make prevalence a function of roost size
df$N=300 #sample size for bats sampled
ggplot(df,aes(x=tRS,y=prev,color=site))+theme_few()+ #plot of data
geom_point()+
scale_x_continuous(trans="log10")
#Fit model with random effect for site: no significant effect of roost size
f1=lme4::glmer(prev~log10(tRS)+(1|site),family=binomial,weights=N,data=df);summary(f1)
#Fit model without random effect for site; roost size is highly significant
f1=glm(prev~log10(tRS),family=binomial,weights=N,data=df);summary(f1)
```

>>> As suggested, we have removed the random effects from the models, including "Site," to avoid redundancy and better assess the effect of roost size (logSize). Despite this adjustment, our conclusions remain unchanged: logSize remains non-significant in all models except for the herpesvirus model. This is likely due to the negative correlation being driven by the low HSV prevalence in juveniles (the period when roost sizes were highest), as noted in the manuscript. Importantly, this correlation disappeared when juveniles were excluded from the model.
The removal of random effects did not alter the overall results, further supporting our decision to exclude them from the final models for the sake of clarity and parsimony.

#R2.35: L209 Report the P-value. Don't write <0.01.
>>> We have modified the presentation of the p-values throughout the entire manuscript.

#R2.36: L210-12 I think you've misinterpreted this analysis. The negative correlation with roost size arises because roost sizes are highest just after birth when juveniles aren't yet infected. It's not because of low prevalence of HSV in juveniles – you already have Age in model M3 so that accounts for age differences. It's because you are examining a dynamic pattern in a static way that doesn't account for the fact that juveniles become infected as they are also dying/leaving colonies so there is a negative correlation b/w roost size and infection even though there may be no actual biological relationship between the two variables.
The analyses would be much informative if you were modeling the change in infection over time and included roost size (i.e. density dependent transmission) in that analysis. See, for example, ref 11.
>>> We believe we are expressing the same idea as the reviewer: that the large roosts and the absence of HSV infection in juveniles are correlated, as they both occur simultaneously. The lack of this correlation when juveniles were excluded from the model supports this hypothesis. We have revised the text for more clarity (page 10: lines 216-220). While we agree that modeling the change in infection over time with roost size could provide valuable insights, we have chosen not to pursue this analysis at this stage, and will consider it for future investigations (see our response to comment #R2.5.).

#R2.37: Table S1 The font in this table is way too small. Make it bigger.
>>> We have reworked all the supplemental tables to now provide better visibility of the statistical details for the models, and we believe this resolves the issue.

#R2.38: Table S1 How can a random effect be "significant"? (i.e. Site in M2). What is this based on?
>>> There is no more random effect in the models (see Tables S1-S5).

#R2.39: L214 What is "sexual segregation level"? Why would you expect prevalence to vary with sex ratio? Does it vary by sex?
>>> The SSAS (Sexual Segregation and Aggregation Statistic) provides a measure of the spatial distribution of males and females across roosts. Specifically, it compares observed patterns of aggregation and segregation to a random distribution of males and females among roosts. In a previous paper (Aguillon et al. 2023), we demonstrated that SSAS varies temporally, with pronounced sexual segregation during the summer months and a more random distribution during the winter. In the present study, prevalence varied by sex for PMV and HSV, and we hypothesized that spatial distribution of sexes (measured via SSAS) could also contribute to prevalence patterns. We therefore included SSAS in the models (pages 23-24: lines 606-612), and removed sex-ratio, as we previously showed that SSAS was correlated to sex-ratio. This decision allows us to focus on the role of spatial distribution in shaping prevalence patterns while avoiding redundancy in the analysis. We hope this clarifies the rationale and relevance of using SSAS and its relationship with sex-specific prevalence patterns.

Aguillon S, et al. 2023. A population in perpetual motion: Highly dynamic roosting behavior of a tropical island endemic bat. Ecol. Evol. 13: e9814.

#R2.40: L229 Show this in a figure. You could do this in Fig 2A by showing adult prev by sex.
>>> We have changed the figure accordingly and now present the prevalence by sex (see Figure 3).

#R2.41: L237 If the difference is not significant then you should not comment on the difference. If you flip a coin 10 times and get heads 6 times you wouldn't say the coin trends towards more heads than tails. Also, you need to show the full statistical analysis for this model as for models above.
>>> We have removed the sentence and added details of the model in supplementary materials (Tables S1 to S5).

#R2.42: L250/Fig 3 Can you show the expected patterns of coinfection based on the prevalence of each of the three pathogens? Given the extremely high prevalence of HSV and moderate prevalences of Lepto and PMV we'd expect a large amount of coinfection.
>>> We considered your suggestion and explored the expected patterns of coinfection based on the individual prevalence of each pathogen. While we generated a preliminary graph to visualize this (see below), we were not convinced of its added value to the manuscript, as the observed high rates of coinfection are already well illustrated in the Venn diagram (Figure S2). For this reason, we decided not to include the additional figure in the main text.

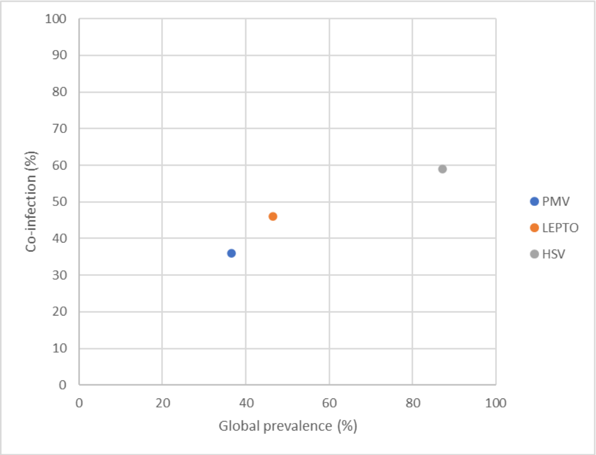

#R2.43: L253-5 The grammar of this sentence needs extensive revision.
>>> We have revised the sentence to improve clarity. In doing so, we have also addressed the distinction between virus-virus and virus-bacteria interactions, as suggested in comment #R1.3 (page 12: lines 272-276).

#R2.44: L259 Tukey, not Tuckey
>>> Corrected.

#R2.45: L290-1 I don't understand this sentence and it seems to contradict the one before it.
>>> We have modified the sentence to make it clearer (page 13: lines 317-320).

#R2.46: L303 You've actually shown extremely little data on "circulation". Patterns of infection are not patterns of acquiring infection. Only your multinomial analyses address circulation and so little attention was paid to them that it's not clear what can be concluded.
>>> To avoid confusion, we replaced the term 'circulation' by 'shedding' (page 14: line 330).

#R2.47: L306 "consistent" not "coherent"
>>> The sentence has been revised (page 14: lines 335-337).

#R2.48: L314 No, this is because you didn't do the analyses properly. See above.
>>> We have revised the sentence to clarify our discussion, now focusing specifically on the effect of roost size, in line with comment #R1.9. Following the revisions of our models, we found that roost size is not a significant variable. We have included contextual elements suggesting that roost size may not be a major factor influencing prevalence in Reunion free-tailed bats, based on the available data and supporting literature (page 14: lines 342-346).

#R2.49: L316-9 This doesn't make any sense. If high risk individuals roosted in high density areas you WOULD see a correlation with density.
>>> With the revisions made to restructure the discussion, this sentence has been removed.

#R2.50: L319-20 Actually you did find evidence of synchrony. Moderately strong for one pathogen, but less so for others. The reason for this is likely the very short time series of your study (2 years), and not much variability in prevalence. In short you'd need much more data to detect synchrony and your vary limited temporal sample cannot be used to suggest synchrony isn't present.
>>> We have revised and enhanced our analysis of synchrony by incorporating new statistical methods, allowing us to detect some degree of synchrony (see response to comment #R2.4).

Furthermore, the limitations of our data in detecting synchrony are now explicitly discussed (page 15: lines 350-354).

#R2.51: L321-4 Revise this after you've done the analyses properly.
>>> We believe that our results support that transient epidemics are not a major driver of PMV, LEPTO and HSV shedding dynamics, but that intrinsic biological cycles rather influence the shedding of persistent/latent infections. We have revised the discussion section for greater clarity (page 15: lines 354-358).

#R2.52: L333 Why "almost"? I thought 100% of HSV+ bats were + on recapture.
>>> You are right, we have adjusted the phrasing accordingly (page 16: lines 398-400).

#R2.53: L339 You have no data to determine if bats clear the pathogen and become reinfected or simply stop shedding and then start shedding again (recrudescence). Revise this sentence accordingly.
>>> We have modified the sentence accordingly (page 17: lines 415-428).

#R2.54: Figure 4C This is the least informative way to show this data and Figure 4 B adds no information and should be removed. In 4C show the 0s and 1s which will provide the same information as the rug plot. In addition, bin the data either into equal sample size bins or roughly equal time intervals and show the prob of that transition (or lack thereof) and SE/CI for each point. This will allow the reader to actually see the data the model is based on.
>> The Figure 4B was intended to illustrate that bats remained positive not only over short periods (multiple captures within six months) but also over longer durations (spanning more than a year). We recognize that this information may not be essential in the main text, so we have moved it to the supplementary information (now Figure S4).

Regarding Figure 4C (now Figure 5B and C): We believe there may have been a misunderstanding in the reviewer's suggestion to "show the 0s and 1s," as the data in this figure are not binary. Instead, they fall into four categories, and the figure presents the proportions of these four categories. Nevertheless, to address the reviewer's concerns and make the data more interpretable, we have incorporated the raw data into the figure. Specifically, we binned the data into intervals of 100 days and plotted points with bars representing 95% confidence intervals. This addition allows readers to directly observe the raw data alongside the model output (represented by a line and shaded area for the model's confidence intervals).

#R2.55: Figure S2 The %s here don't add up to 100 so the figure legend should indicate these are the percentages of bats infected with at least one pathogen and the remainder (give the %) were not shedding any of the 3 pathogens. For each section of the venn diagram please add the expected % given the prevalence for each of the three pathogens. My quick calculations are not consistent with the analyses in the paper.
>>> We have revised the legend to explicitly indicate that the percentages represent bats tested for the three infectious agents (n=3784). Moreover, the percentage of bats that were not shedding (10.54%) is now indicated on the diagram (Fig S2), ensuring the total sums to 100%. We calculated the expected probabilities for each section of the Venn diagram based on the individual prevalence of the three pathogens, assuming independence between shedding events. These expected values have been added to the diagram for comparison with the observed data, providing additional context for the results.

#R2.56: Fig S3 The violin plots on this figure make no sense. They extend to negative time intervals. Please correct. Instead of showing nonsensical violin plots why not show the mean and SE/CI?
>>> We have modified the figure and now present boxplots.

#R2.57: L350-4 There are multiple empirical papers showing viral recrudescence that should be cited instead of the papers cited here (the modeling study and hypothesis paper, refs 58, 63). Your Ref 11 (Epstein et al) and these two other papers show strong evidence of recrudescence: A. R. Sohayati et al.; Henipavirus Ecology Research Group, Evidence for Nipah virus recrudescence and serological patterns of captive Pteropus vampyrus. Epidemiol. Infect. 139, 1570–1579 (2011).
A. J. Peel et al., Henipavirus neutralising antibodies in an isolated island population of African fruit bats. PLoS One 7, e30346 (2012).
>>> The suggested references (Epstein et al. 2020, Sohayati et al. 2011, Peel et al. 2012) are already cited in the preceding sentence (refs 11, 60, 62), which discusses long-term continuous and intermittent viral shedding. To address the comment and highlight recrudescence more explicitly, we have revised the sentence to emphasize this phenomenon (page 17: lines 426-433).

#R2.58: L364-9 The absence of a pattern for HSV is likely because prevalence is so high. You don't need to claim that the effect of reproduction on shedding is "variable" because you didn't find an effect for HSV.
>>> The paragraph has been modified accordingly (page 18: lines 452-453).

#R2.59: L380 This sentence contradicts the one before it.
>>> In this section, we addressed the possibility that persistent infections might reactivate during pregnancy and mating periods. However, we also discussed alternative mechanisms, such as the immigration of infected individuals during large aggregations of bats associated with pregnancy periods. To resolve any ambiguity or apparent contradiction, we have rephrased this part of the discussion for improved clarity (pages 18: lines 461-465, page 19: lines 472-475).

#R2.60: L383 You've just shown data that support the opposite of this.
>>> We are unsure which statement and data the reviewer is referring to, but page 18, line 463 mentions that it "*could benefit the mothers through an immune boost [94].*" Here, we propose that the reactivation of shedding during pregnancy may be linked to immunosuppression (to protect the fetus). This reactivation could then enhance the individual's immunity and contribute to the long-term maintenance of immune responses. This part of the discussion has been modified to make it clearer (page 18: lines 461-464).

#R2.61: L388 This seems to be more likely to be an age effect than an effect of reproduction. Since shedding prevalence is 5-10x lower for juveniles it's likely that the non-reproductive individuals are likely just younger and the effect may be unrelated to reproduction. The same may be the case for females.
>>> To address this potential bias, we have acknowledged and discussed it in multiple sections of the manuscript. For a detailed explanation, please see our response to comment #R2.9.

#R2.62: L429 You didn't measure infection. You measured pathogen shedding. Revise the wording here and throughout the manuscript.

>>> The wording has been revised throughout the manuscript to accurately reflect that we measured pathogen shedding rather than infection.

#R2.63: L431 Antibody levels likely have nothing to do with shedding. HSV often hides out in nerves where antibodies are ineffective. It's likely that tissue tropism rather than antibody levels that likely explain HSV prevalence.
>>> We agree with the reviewer and have removed this section of the discussion, in line with other comments (#R1.1 and #R2.65) regarding this part of the manuscript.

#R2.64: L436 It's clearly not "quasi-total" since prevalence wasn't 0.
>>> We have removed this section of the discussion.

#R2.65: L422-442 You have no data on antibodies so I don't understand why you are speculating so wildly about it. Please remove this entire section of text and focus on the data you have actually collected.
>>> We have removed this paragraph.

#R2.66: L456-8 This isn't consistent with Fig S2. This figure appears to show that
>>> It seems that a portion of the reviewer's comment is missing at the end of the sentence. Based on the context provided, we have carefully reviewed the relevant section and revised our discussion accordingly (page 19: lines 486-488). The updated text aligns with the results from the revised models (see page 23: lines 605-606).

#R2.67: L469 You have NOT shown latent herpes virus infection. You've shown the opposite. Not a single bat that was shedding upon first capture was not shedding later. This means you have evidence against latent infections for HSV. Revise throughout the paper.
>>> We believe our data may support a model of latent HSV infection with periodic reactivation. Notably, Griffiths et al. 2023 recently demonstrated that the active shedding phase can be particularly prolonged compared to the latency phase in *Desmodus rotundus* infected with a betaherpesvirus. Our findings align with such a mechanism, although continuous shedding cannot be ruled out. We have updated the discussion accordingly and incorporated additional sequencing results to discuss this aspect (page 16: lines 400-408).

Griffiths et al. 2023. Inferring the disruption of rabies circulation in vampire bat populations using a betaherpesvirus-vectored transmissible vaccine. PNAS 120: e2216667120.

#R2.68: L471-2 Revise after doing the analyses properly.
>>> This sentence has been removed with the revision of the discussion.

#R2.69: L473 You have no data on this. Remove.
>>> This has been removed.

#R2.70: L518 What pathogens do these primers detect? A single virus for HSV and PMV? Multiple viruses? This is hugely important for interpreting patterns of shedding. Also, is there a reason you didn't use qPCR? Quantitative estimates of shedding would have been extremely valuable.
>>> Please see our response to comment #R.1.1. We now addressed this point in both the methods and discussion. We actually did use qPCR for Leptospira and now present the results on age- and reproduction-related effects on the relative quantity of bacteria shed in urine (page 10: lines 229-233 and page 12: lines 263-264).

#R2.71: L544 These analyses are highly problematic. See comments above.
>>> We have carefully reviewed the models with all the comments above.

#R2.72: L575 What data were used to assess segregation? I didn't see any description of how you measured this.
>>> We have provided additional details in the revised manuscript (pages 23-24: lines 606-612) regarding how sexual segregation was assessed, in line with the methodology used in Aguillon et al. (2023). Specifically, we used capture data to evaluate the spatial distribution of males and females across different roosts during each sampling period.

Aguillon S, et al. 2023. A population in perpetual motion: Highly dynamic roosting behavior of a tropical island endemic bat. Ecol. Evol. 13: e9814.

#R2.73: L578 This is an incorrect way to assess this. Why not include it in your GAMMs?
>>> We have now included the sexual segregation parameter in the GAMs (pages 23-24: lines 606-612 and see Tables S1 and S3).

#R2.74: L584 Spearman tests have very poor power. It's no wonder you found weak evidence for synchrony. Why not use a more powerful test?
>>> We have switched to using Pearson's correlation tests (page 22: lines 575-576). Please refer to our response to comment #R2.4 for further details.

#R2.75: L601 Rstudio is simply a GUI for R. Cite the version of R you used instead.
>>> Changed (page 25: line 646).
