# Supplementary material for: Role of individual and population heterogeneity in shaping dynamics of multi-pathogen shedding in an island endemic bat
Source: PLoS Pathog. 2025 Jul 11;21(7):e1013334. doi: 10.1371/journal.ppat.1013334 (PMC12273948; doi:10.1371/journal.ppat.1013334)
Supplement: Attachment — Submitted filename: PPATHOGENS-D-24-01674R2_response.pdf [file ppat.1013334.s014.pdf]

Samantha Aguillon
UMR Processus Infectieux en Milieu Insulaire Tropical
2, rue Maxime Rivière 97490 Sainte Clotilde
Reunion Island
Email : samantha.aguillonfr@gmail.com

Sainte Clotilde, 14 June 2025

Dear Editors,

Please find with this letter, the revised version of our manuscript entitled "**Role of individual and population heterogeneity in shaping dynamics of multi-pathogen shedding in an island endemic bat**". We have carefully considered all remarks and modified the manuscript accordingly. You will find the reviewer's comments and our replies at the end of this letter.

All authors have participated in revising the manuscript and have agreed on its resubmission to *Plos Pathogens.*

Thank you for your consideration of our work.

Sincerely yours,
Samantha Aguillon, on behalf of the other authors

Reviews of Manuscript : PPATHOGENS-D-24-01674R2

Please note that author's responses follow each reviewer's comment and start with ">>>". Line numbers correspond to the clean version of the manuscript.

Journal Requirements:

1) Regarding Figure 1, thank you for stating "Maps were created using Qgis [111] with a background of OpenStreetMap France under License CC- BY- SA 2.0."

Please provide a direct link to the base layer of the map (i.e., the country or region border shape) and ensure this is also included in the figure legend.
>>> We added a direct link in the legends of figures 1 and 2.

Part I – Summary

Reviewer #1:
I am satisfied with the authors' responses to my questions, and cannot comment further on the modelling. This really is incredible data. I look forward to seeing future work from this system

Part II – Major Issues: Key Experiments Required for Acceptance

Reviewer #1:
None

Part III – Minor Issues: Editorial and Data Presentation Modifications

Reviewer #1:
#R1.1: This is an informative and thoughtful response. I appreciate the efforts and it is great to hear the exciting work being done. I appreciate the extra text added, however still feel that it's a bit obscured. Perhaps the authors could add a note at the end of the first paragraph of the results or to the caption of Table 1 flagging that detections for each pathogen are broad results family/genus level (as appropriate) and may represent combined prevalences across multiple species.
>>> Added in the Table 1's caption (page 9, lines 190-192).

This may not help the authors at this stage, but regarding the substitution of infection for shedding throughout, I agree with reviewer 2 that it is important to mechanistically consider the difference between the two terms, and interpret the results accordingly. However, I think that co-infection is a widely used term to denote an individual from whom two infectious agents are simultaneously detected. Rarely do any infection studies—in wildlife or humans—go to the effort of distinguishing between infection and shedding. While less can be said about infection status in the absence of detection of shedding, the positive detection of the infectious agent more reliably reflects current or very recent infection. With that in mind, I have no problem with the term co-infection. I think co-

shedding is an unnecessarily overcomplicated alternative and does not help with clarity. Perhaps the editor can provide a view here to guide the authors.
>>> In the context of our study, we believe that the term “shedding” is a more appropriate and mechanistically accurate term than “infection.” This is particularly relevant for bats, where detection of herpesviruses, paramyxoviruses, and *Leptospira* likely reflects episodes of active shedding, which can occur intermittently due to latent infections or recrudescence. The term “shedding” thus more accurately reflects what we detect: the presence of pathogen genetic material in excreted fluids, which may indicate either active infection or reactivation from latency. In this sense, our use of “co-shedding” aims to more precisely reflect the biological processes involved.

I may have added a few additional points that I didn't pick up on the first time, but these are minor.

#R1.2: Line 64: "Reproductive individuals (both during the pregnancy and mating)". Suggest “Reproductively-active individuals (during the pregnancy, lactation and mating periods)”. I am reminded later on that this is clarified in the methods, but this needs to be clear in the abstract too.
>>> As we did not properly test for the effect during the lactation, but focused only on pregnancy and mating, we decided to keep the original sentence replacing “reproductive individuals (both during the pregnancy and mating)” by “reproductively-active individuals (both during the pregnancy and mating)” (page 3, line 64).

#R1.3: Line 65: Should be associated ‘with’ rather than 'to'. This is a repeated grammatical error in the author summary
>>> Changed (page 3, line 65, and page 4, line 79).

#R1.4: Line 65: ‘could’ rather than ‘can’
>>> Changed (page 3, line 66).

#R1.5: Line 101: co-infections are common in bats [16–20]. Also see meta analysis in Jones et al 2023 Viruses
>>> Reference added (page 5, line 101).

#R1.6: Line 206: Roost loadings varied only substantially in both magnitude and direction. This analysis is not in my expertise, but the placing of the word 'only' seems to make this sentence unclear.
>>> The term “only” has been removed (page 10, line 208).

#R1.7: Line 218: "see below"
>>> Changed (page 10, line 221)

#R1.8: Line 252: During pregnancy. For clarity, "During the pregnancy period,..."
>>> Changed (page 11, line 254)

#R1.9: Line 296-298: These two sentences are a little unclear, and I think need restructuring. Either model on the subsequent sentence for HSV, or something like: All captured bats were marked (by tattooing on the wing) allowing to collect shedding data from recaptured bats, and enable comparison of results from samples collected on initial capture and on recapture. XX bats were captured more than once. We tested 471 and 457 samples from XX recaptured bats for PMV and LEPTO, respectively.

>>> We thank the reviewer for the suggestion and clarify the sentences (page 13, lines 299 – 302).

<u>#R1.10:</u> Line 304: Check the y-axis labels in Fig S3B. Should this be time interval?
>>> We agree with the reviewer and corrected the labels.

<u>#R1.11:</u> Line 322: remained non-reproductively active. I got to here and was a bit unclear what this means for both females and males. I see now that it is in your methods below, but it would be helpful if you could briefly define "reproductively active" for males and females e.g. ..reproductively active (pregnant, lactating or post-lactating adult females, or adult males with large testes) ...
>>> We added clarification about the definition of reproductively active individuals in the sentence (page 14, lines 326-327).

<u>#R1.12:</u> Line 396-398: Interestingly in D. rotundus infected with a betaherpesvirus [14] , the length of the active period (when shedding occurs) appears to be longer than the latency state (when shedding resumes). This sentence is unclear. Latency isn't usually associated with shedding. I would have thought active = shedding and latency = no shedding. Also, note that the study being cited is a modelling study - it should be made clear that there is no empirical evidence to support this point, but that it was a feature of the best fitting mechanistic model
>>> We agree with the reviewer and changed the sentence, and also added a clarification about the study cited (page 16, lines 401-406).

<u>#R1.13:</u> Line 399: although we cannot exclude continuous shedding from persistent infections. I think this hypothesis is undersold here. Given that all individuals were shedding on recapture, this should be presented at least as an equal scenario, rather than a caveat
>>> We changed the sentence (pages 16-17, lines 406-407).

<u>#R1.14:</u> Line 416-418: indicating that cycles of clearance followed by reinfection may occur within the bat population, although the possibility of recrudescence from latent infections cannot be ruled out. Similarly, I think these are equally plausible scenarios and should be presented as such
>>> Changed (page 17, lines 425-426).

<u>#R1.15:</u> Line 419: bacteria. because the bacteria?
>>> Changed (page 17, lines 428).

<u>#R1.16:</u> Line 452: Our results suggest that pregnancy increases the probability of PMV and LEPTO shedding, as well as the likelihood of dual and triple shedding in Reunion free - tailed bats. The results suggest that this was clouded by age though, considering M0 females? The nuance of the M0 results was striking to me in the results section, but I think the discussion of those results here is not faithful to them. To me, the results showed clearly that these effects were not reproduction effects but were age effects. Here, the discussion is focussed on the reproduction results, with the age as a small caveat. I really think that the age results should be considered alongside the reproduction results. i.e. while reproduction is often found to be a significant predictor, and many of our results here supported this, our ability to separate non-juvenile individuals into those that had previously bred (reproductive adults) and those who had not (likely younger pre-reproductive individuals), the effects of reproduction disappeared.
>>> We have revised the discussion to better reflect this nuance and to present the age-related hypothesis on an equal footing with the reproduction hypothesis(page 18, lines 474-476).

#R1.17: Line 494: the timing. This is an important point to get across clearly. It took be a couple of reads to follow. Perhaps emphasis this point as 'asynchronous timing'
>>> Changed (page 20, line 505).
